# Efficacy of GLP-1 receptor agonists in Parkinson’s disease: a systematic review and exploratory network meta-analysis of randomized controlled trials

**DOI:** 10.1007/s10072-026-08929-1

**Published:** 2026-04-17

**Authors:** Muaz Ali, Arkansh Sharma, Amith Paruchuri, Faizan Shahzad, Marwah Bintay Khalid, Karthik N. B., Mona Avanaki, Allimuthu Nithyanandam, Tarannum Khan, Vinay Suresh

**Affiliations:** 1 Cleveland Clinic Florida, Weston, FL United States of America; 2 Government Medical College, Omandurar, Chennai, Tamil Nadu India; 3 All India Institute of Medical Sciences, New Delhi, India; 4 Department of Medicine, Rawalpindi Medical University, Rawalpindi, Pakistan; 5 Department of Neurology, Tamil Nadu Government Multi-Super Speciality Hospital, Omandurar Government Estate, Chennai, Tamil Nadu India; 6 University of Oxford, Oxford, UK

**Keywords:** Parkinson’s disease, GLP-1 receptor agonist (GLP-1RA), Quality of life, Cognition

## Abstract

**Background:**

Current therapies for Parkinson’s disease lack proven disease-modifying effects. Glucagon-like peptide-1 receptor agonists (GLP-1RAs), developed for type 2 diabetes, have shown potential neuroprotective properties. Their comparative efficacy in Parkinson’s disease remains unclear.

**Methods:**

A systematic search (inception–February 2026) identified randomized controlled trials evaluating GLP-1RAs in Parkinson’s disease. Pairwise and frequentist random-effects network meta-analyses were performed. The primary outcome was MDS-UPDRS Part III (ON state).

**Results:**

Five trials (*n* = 708) were included. Pairwise meta-analysis showed no significant overall improvement in MDS-UPDRS Part III (MD –2.00; 95% CI –5.46 to 1.46; I² = 80.5%). Network meta-analysis demonstrated significant ON-state motor improvement with Exenatide 20 µg/day (MD –9.80; 95% CI –14.47 to –5.13) and Lixisenatide 20 µg/day (MD –3.08; 95% CI –5.31 to –0.85). No significant effects were observed for MDS-UPDRS Part III (OFF-state) or other domains (Parts I, II, and IV; ON-state). NLY01 at 5 mg/week improved PDQ-39, while NMSS showed dose-dependent divergence. Gastrointestinal adverse events were more frequent with GLP-1RAs.

**Conclusion:**

GLP-1 receptor agonists may provide dose-specific motor benefits in Parkinson’s disease, but evidence for broader clinical improvement is limited. Larger, longer-duration trials are needed.

**Supplementary Information:**

The online version contains supplementary material available at 10.1007/s10072-026-08929-1.

## Introduction

Parkinson’s disease (PD) is a progressive neurodegenerative disorder marked by both motor and non-motor symptoms (1). Global estimates and projections indicate a steep rise in prevalence over the coming decades with its impact not only clinical but also economic and social, due to healthcare costs, disability, and long-term caregiver needs (2).

Pathologically, PD is defined by progressive loss of melanin-containing dopaminergic neurons in the substantia nigra and intracellular accumulation of α-synuclein (1). The disease is considered multifactorial, arising from interactions between genetic susceptibility and environmental exposures (3). Key mechanisms include oxidative stress, mitochondrial dysfunction, abnormal protein processing, and disrupted cellular energy mechanisms (3). Increasing evidence links PD with type 2 diabetes mellitus, with shared pathways such as insulin resistance, inflammation, and mitochondrial injury (4). This overlap has led to interest in glucagon-like peptide-1 receptor agonists (GLP-1RAs), a class of drugs originally developed for diabetes treatment (4). These agents enhance glucose-dependent insulin secretion and suppress glucagon release, while also showing potential neuroprotective effects in experimental models (4). In a 6-OHDA rat model, Jalewa et al. showed that GLP-1/GIP receptor agonism may reduce neuroinflammation and dopaminergic neuronal loss, and improve dopaminergic function (5). Early clinical trials of agents such as exenatide, and liraglutide report modest motor benefits and possible disease-modifying effects, but most studies are small and require validation (6).

As current evidence is limited and involves multiple competing agents, simple pairwise meta-analysis is insufficient for comparison. Therefore, to enable both direct and indirect comparisons, this study undertakes a pairwise meta-analysis alongside an updated exploratory network meta-analysis of randomized trials to compare the effectiveness of individual GLP-1 receptor agonists in Parkinson’s disease.

## Methods

### Literature search

This systematic review and network meta-analysis was registered prospectively with the PROSPERO International Prospective Register of Systematic Reviews under the registration ID CRD420251109255. The review process adhered strictly to the PRISMA 2020 guidelines and the methodological framework outlined in the Cochrane Handbook for Systematic Reviews of Interventions. As the study involved secondary analysis of published data, no ethical approval or patient consent was necessary.

A comprehensive literature search was performed in four electronic databases, PubMed, EMBASE, Scopus, and Web of Science from inception through February 2026. The search targeted randomized controlled trials evaluating GLP-1 receptor agonists in patients with Parkinson’s disease, using a combination of controlled vocabulary and keyword terms related to GLP-1 agents and Parkinsonian disorders. Database-specific search formats were applied. Reference lists of eligible articles and relevant reviews were also screened to ensure completeness. The detailed search strategy is provided in Supplementary Material.

### Inclusion and exclusion criteria

Randomized controlled trials (RCTs) were deemed eligible if they enrolled adult patients with a clinical diagnosis of Parkinson’s disease and investigated the efficacy of a GLP-1 receptor agonist, administered either as monotherapy or in combination with standard antiparkinsonian therapy. To qualify for inclusion, studies were required to report at least one of the predefined outcomes of interest, namely the Movement Disorder Society Unified Parkinson’s Disease Rating Scale (MDS-UPDRS) subparts I–IV, the Parkinson’s Disease Questionnaire-39 (PDQ-39), or the Non-Motor Symptoms Scale (NMSS). These outcome measures were selected appropriately to comprehensively capture both motor and non-motor domains, including functional disability, quality of life, cognitive performance, and neuropsychiatric manifestations.

The primary outcome of this review was the MDS-UPDRS Part III (motor examination) score. All other outcomes, including MDS-UPDRS subparts I, II, and IV, PDQ-39, and NMSS, were considered secondary outcomes.

To maintain methodological rigor, we excluded commentaries, letters to the editor, editorials, narrative or systematic reviews, observational studies, case series, short communications, conference abstracts, and preprints, as only fully published RCTs that had undergone formal peer review were considered eligible for inclusion.

### Data extraction

Two independent reviewers conducted a two-stage screening process, first assessing titles and abstracts and then reviewing full texts. Disagreements were resolved through consensus or consultation with a third reviewer.

A standardized data extraction form was used to collect study-level information, including first author, publication year, Parkinson’s disease diagnostic criteria, mean age, female-to-male ratio, baseline MDS-UPDRS Parts I–IV scores, Total PDQ-39 scores, Total NMSS scores, Hoehn and Yahr stage, type and dose of GLP-1 receptor agonist, treatment duration, follow-up length, and sample size per arm. Outcome data were extracted as means and standard deviations for each scale, along with the total number of participants per treatment group. When multiple follow-up time points were reported, the longest available follow-up was prioritized to ensure consistency in effect estimation.

### Statistical analysis

All analyses were conducted in R (v4.5.2) using the netmeta and meta packages. A frequentist network meta-analysis within a graph-theoretical framework was performed to combine direct and indirect evidence. Random-effects models were prespecified, with between-study variance (τ²) estimated using restricted maximum likelihood (REML). Placebo/control was the reference comparator. Continuous outcomes were expressed as mean differences (MDs) with 95% confidence intervals (CIs), applying the Hartung–Knapp adjustment to improve inference in small samples. Treatment rankings were estimated using P-scores.

Transitivity was evaluated by comparing potential effect modifiers across treatment comparisons, including mean age, proportion of males, baseline MDS-UPDRS Parts I–IV ON, and PDQ-39 scores. Conventional pairwise random-effects meta-analyses (inverse-variance method; REML estimator) were also conducted for comparisons with direct evidence. Heterogeneity was assessed using Cochran’s Q and quantified with I².

### Quality assessment

The methodological quality of the included randomized controlled trials was evaluated using the Cochrane Risk of Bias 2.0 (RoB 2.0) tool. This tool assesses five domains of bias: bias arising from the randomization process, deviations from intended interventions, missing outcome data, measurement of the outcome, and selection of the reported result. Each domain was rated as low, some concerns, or high risk of bias. Judgments were independently made by two reviewers, with disagreements resolved through discussion. Overall quality ratings were used to inform interpretation of treatment effect certainty.

## Results

**Fig. 1:**
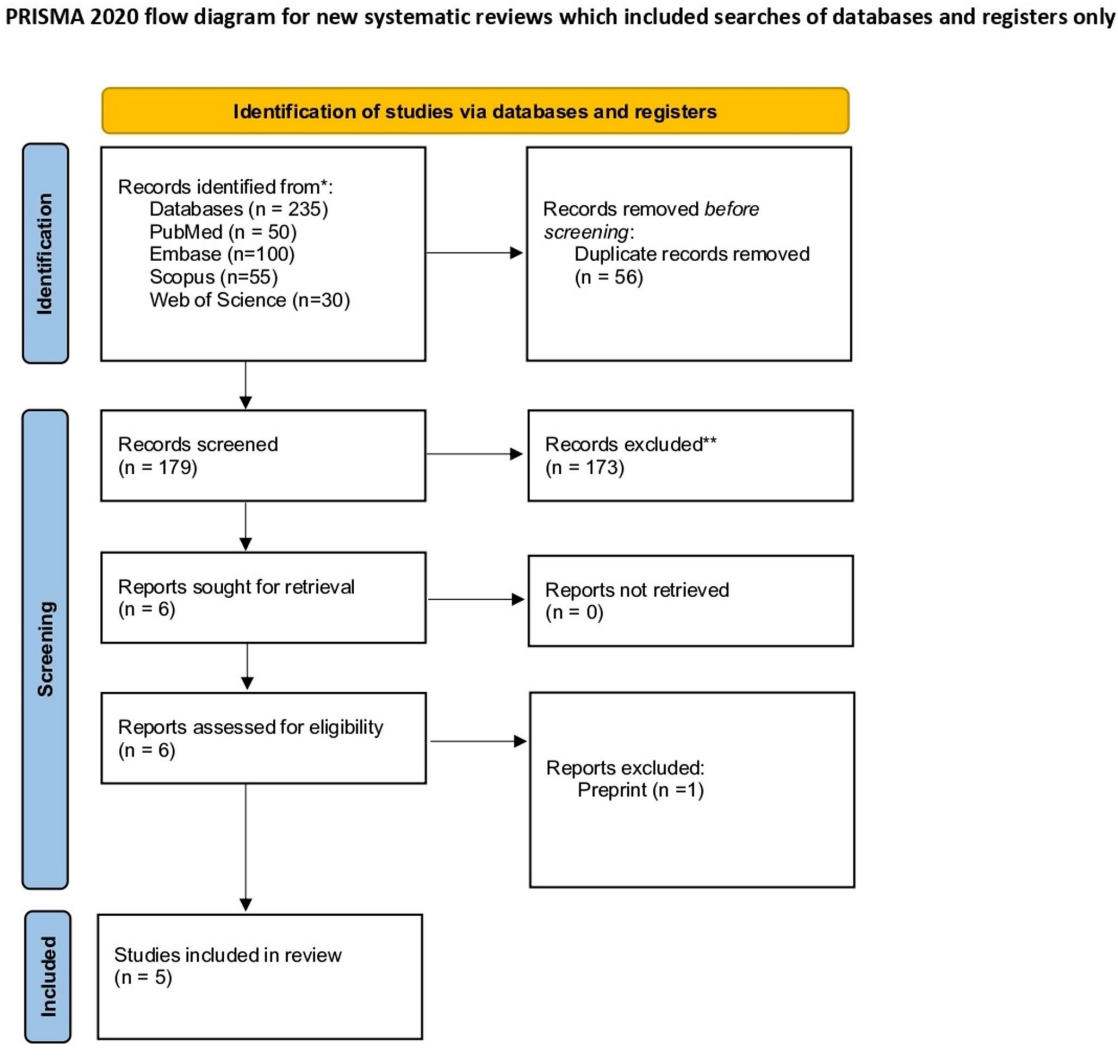
PRISMA flow chart

A total of 235 records were identified through database searches: PubMed (*n* = 50), EMBASE (*n* = 100), Scopus (*n* = 55), and Web of Science (*n* = 30). After removing 56 duplicates, 179 records remained for screening. Title and abstract screening excluded 173 records. Six full-text articles were retrieved and assessed for eligibility, all successfully obtained. One study was excluded because it was a preprint. Ultimately, five randomized controlled trials met the inclusion criteria and were included in the final systematic review. The study selection process is summarized in Fig. 1; Table 1.

**Table 1:** Characteristics of included studies and participants

Author Year	Group	Sample Size	Age Mean (SD)	Gender Male (%)	MDS-UPDRS I Mean (SD)	MDS-UPDRS II Mean (SD)	MDS-UPDRS III Mean (SD)	MDS-UPDRS III On-med Mean (SD)	MDS-UPDRS III Off-med Mean (SD)	MDS-UPDRS IV Mean (SD)	PDQ-39 Mean (SD)	NMSS Mean (SD)	Intervention Method	Follow-up	Hoehn and Yahr Stage	LED (Mean ± SD or Median [IQR])	Parkinson’s Diagnostic Criteria	Primary End Point
Athauda 2017 (13)	Exenatide	31	61.6 (8.2)	22 (71%)	9.8 (4.8)	12.5 (6.7)	32.8 (9.7)	19.4 (8.4)	4.7 (3.1)	19.9 (13.7)	19.9 (13.7)	24.6 (19.8)	2 mg once weekly for 48 weeks	48w	≤ 2.5	773.9 (260.9)	UK Brain Bank criteria	MDS-UPDRS III OFF
Athauda 2017 (13)	Control	29	57.8 (8.0)	22 (76%)	9.2 (3.8)	10.7 (5.3)	27.1 (10.3)	14.4 (8.2)	5.3 (3.0)	21.1 (13.0)	21.1(13.0)	28.3 (24.7)	Placebo once weekly for 48 weeks (Placebo comparator)	48w	≤ 2.5	825.7 (215.0)	UK Brain Bank criteria	MDS-UPDRS III OFF
Aviles-Olmos 2013 (14)	Exenatide	20	61.4 (6)	15 (75%)	10.4 (4.1)	10.2 (5.2)	31 (11.2)	23.5 (6.3)	6.3 (2.4)	19.2 (13.5)	19.2 (13.5)	N/A	Initially 5 µg/day for 1 month, then 20 µg/day for remainder of 12 months	12 m	2–2.5	973 (454)	UK Brain Bank criteria	MDS-UPDRS III OFF
Aviles-Olmos 2013 (14)	Control	24	59.4 (8.4)	20 (83%)	11.6 (4.7)	12.9 (6.2)	34 (16.1)	25.3 (10.7)	6.3 (3.4)	24.5 (12.8)	24.5 (12.8)	N/A	Conventional PD treatment (control)	12 m	2–2.5	977 (493)	UK Brain Bank criteria	MDS-UPDRS III OFF
McGarry 2024 (15)	NLY01 (5 mg)	85	60.6 (10.0)	54 (64%)	4.0 (3.7)	5.0 (4.1)	N/A	22 (8.2)	N/A	N/A	N/A	N/A	NLY01 5 mg once weekly for 36 weeks	36w	≤ 2.5	N/A	UK Brain Bank criteria	Sum MDS-UPDRS II + III
McGarry 2024 (15)	NLY01 (2.5 mg)	85	62.1 (9.0)	60 (71%)	4.2 (3.1)	4.8 (3.6)	N/A	22.7 (8.1)	N/A	N/A	N/A	N/A	NLY01 2.5 mg once weekly for 36 weeks	36w	≤ 2.5	N/A	UK Brain Bank criteria	Sum MDS-UPDRS II + III
McGarry 2024 (15)	Control	84	61.8 (8.1)	52 (62%)	4.7 (4.2)	4.9 (3.6)	N/A	22.3 (9.1)	N/A	N/A	N/A	N/A	Placebo once weekly for 36 weeks	36w	≤ 2.5	N/A	UK Brain Bank criteria	Sum MDS-UPDRS II + III
Meissner 2024 (16)	Lixisenatide	78	59.5 (8.1)	44 (56%)	6.1 (4)	5 (3.5)	N/A	14.8 (7.3)	0.3 (1.3)	17.4 (10.9)	17.4 (10.9)	N/A	Initially 10 µg/day for 14 days, then 20 µg/day for remainder of 12 months	12 m	< 3	317 (179)	UK Brain Bank criteria	MDS-UPDRS III ON
Meissner 2024 (16)	Control	78	59.9 (8.4)	48 (62%)	6.4 (4.2)	5.4 (4.3)	N/A	15.5 (7.8)	0.2 (0.8)	16.8 (13)	16.8 (13)	N/A	Placebo daily for 12 months	12 m	< 3	355 (215)	UK Brain Bank criteria	MDS-UPDRS III ON
Vijiaratnam 2025 (9)	Exenatide	97	61.02 (9.05)	69 (71%)	7.9 (4.9)	7.4 (4.9)	N/A	32.2 (12.5)	20.0 (10.2)	3.9 (3.3)	11.9 (9.3)	30.5 (24.9)	2 mg once weekly for 96 weeks	96w	≤ 2.5	475 (340–615)	Queen Square Brain Bank criteria	MDS-UPDRS III OFF
Vijiaratnam 2025(9)	Control	97	60.35 (9.26)	69 (71%)	7.5 (4.8)	7.5 (4.9)	N/A	32.3 (13.3)	20.8 (10.4)	3.8 (3.2)	10.6 (8.0)	28.2 (24.0)	Placebo once weekly for 96 weeks	96w	≤ 2.5	475 (300–700)	Queen Square Brain Bank criteria	MDS-UPDRS III OFF

### Pairwise meta analysis result

#### MDS-UPDRS part III (ON state)

**Fig. 2:**
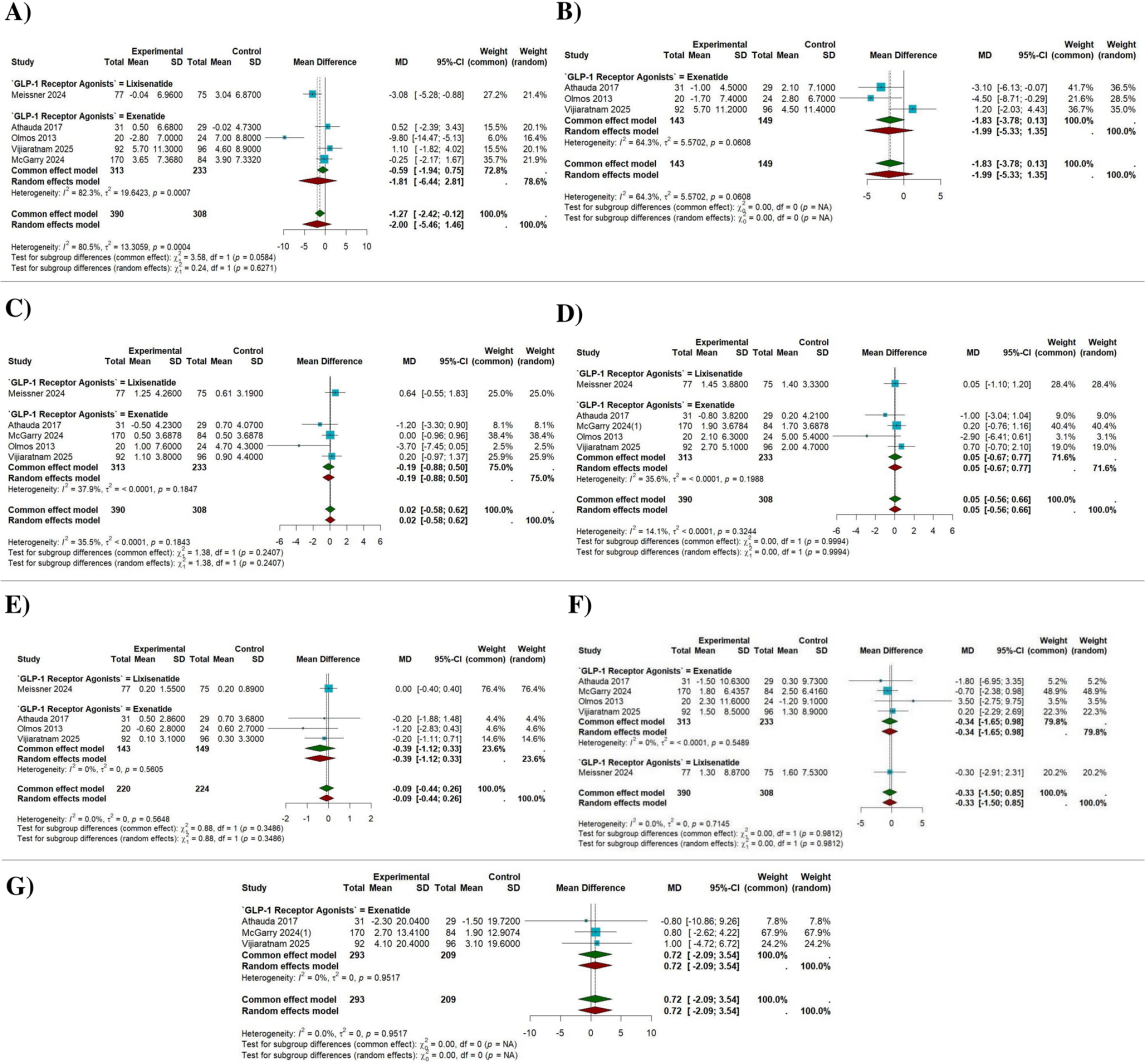
**A** Pairwise meta analysis forest plot of MDS-UPDRS part III (ON State). **B** Pairwise meta analysis forest plot of MDS-UPDRS part III (OFF State). **C** Pairwise meta analysis forest plot of MDS-UPDRS part I (ON State). **D** Pairwise meta analysis forest plot of MDS-UPDRS part II (ON State). **E** Pairwise meta analysis forest plot of MDS-UPDRS part IV (ON State). **F** Pairwise meta analysis forest plot of PDQ-39. **G** Pairwise meta analysis forest plot of NMSS

Five studies including 708 participants (396 intervention, 312 control) evaluated motor outcomes in the ON state using the MDS-UPDRS Part III. Under the random-effects model, GLP-1 receptor agonists demonstrated a pooled mean difference (MD) of − 2.00 (95% CI − 5.46 to 1.46; *p* = 0.258). Although the point estimate favored treatment, the effect was not statistically significant. Substantial heterogeneity was observed (I² = 80.5%; τ² = 13.31), with a significant Q test (Q = 20.49, *p* = 0.0004), indicating considerable variability across studies. In subgroup analysis, Lixisenatide (1 study) showed a statistically significant reduction in motor scores (MD − 3.08; 95% CI − 5.28 to − 0.88). In contrast, Exenatide (4 studies) demonstrated a non-significant effect (MD − 1.83; 95% CI − 3.78 to 0.13; I² = 82.3%; τ² = 19.64). There was no statistically significant difference between subgroups (Q = 0.24, *p* = 0.627) (Fig. 2A).

#### MDS-UPDRS part III (OFF state)

Three studies including 292 participants (143 intervention, 149 control) evaluating exenatide in the OFF-medication state were pooled using a random-effects model. Motor outcomes were assessed under a standardized, practically defined OFF condition, with levodopa withheld overnight (≥ 8 h) and long-acting dopaminergic medications for 24–36 h. Assessments, including MDS-UPDRS Part III, were performed in the early morning before resumption of usual therapy, ensuring comparability across studies. The pooled mean difference was − 1.99 (95% CI − 5.33 to 1.35; *p* = 0.242), indicating no statistically significant difference, although a trend favoring exenatide was observed. Moderate heterogeneity was present (I² = 64.3%; τ² = 5.57) (Fig. 2B).

#### MDS-UPDRS part I (ON state)

Five studies (*n* = 708; 396 intervention, 312 control) assessed non-motor experiences of daily living (Part I, ON state). The pooled analysis showed no significant effect of GLP-1 receptor agonists (MD 0.02; 95% CI − 0.58 to 0.62; *p* = 0.945), with the estimate close to zero. Heterogeneity was low to moderate (I² = 35.5%; τ² < 0.0001), and the Q test was not significant (Q = 6.21, *p* = 0.184). Subgroup analysis revealed no significant effects for Lixisenatide (MD 0.64; 95% CI − 0.55 to 1.83) or Exenatide (MD − 0.19; 95% CI − 0.88 to 0.50; τ² < 0.0001). There was no significant difference between subgroups (Q = 1.38, *p* = 0.241) (Fig. 2C).

#### MDS-UPDRS part II (ON state)

Five studies (*n* = 708; 396 intervention, 312 control) were included. The pooled MD was 0.05 (95% CI − 0.56 to 0.66; *p* = 0.874), indicating no significant difference between treatment and control groups. Heterogeneity was low (I² = 14.1%; τ² < 0.0001), with a non-significant Q test (Q = 4.66, *p* = 0.324). Subgroup analyses showed no significant effects for Lixisenatide (MD 0.05; 95% CI − 1.10 to 1.20) or Exenatide (MD 0.05; 95% CI − 0.67 to 0.77; τ² < 0.0001). No subgroup differences were observed (Q = 0.00, *p* = 0.999) (Fig. 2D).

#### MDS-UPDRS part IV (ON state)

Four studies (*n* = 444; 220 intervention, 224 control) assessed motor complications (Part IV, ON state). The pooled MD was − 0.09 (95% CI − 0.44 to 0.26; *p* = 0.602), demonstrating no significant treatment effect. No heterogeneity was observed (I² = 0.0%; τ² = 0), and the Q test was non-significant (Q = 2.04, *p* = 0.565), indicating highly consistent findings. Subgroup analysis showed no effect for Lixisenatide (MD 0.00; 95% CI − 0.40 to 0.40) and a non-significant reduction for Exenatide (MD − 0.39; 95% CI − 1.12 to 0.33; τ² = 0). There was no significant subgroup difference (Q = 0.88, *p* = 0.349) (Fig. 2E).

#### PDQ-39

Five studies (*n* = 708; 396 intervention, 312 control) evaluated quality of life using the PDQ-39. The pooled MD was − 0.33 (95% CI − 1.50 to 0.85; *p* = 0.584), indicating no statistically significant difference between groups. No heterogeneity was detected (I² = 0.0%; τ² = 0), with a non-significant Q test (Q = 2.12, *p* = 0.715). Subgroup analyses showed non-significant effects for Exenatide (MD − 0.34; 95% CI − 1.65 to 0.98; τ² < 0.0001) and Lixisenatide (MD − 0.30; 95% CI − 2.91 to 2.31), with no subgroup difference (Q = 0.00, *p* = 0.981) (Fig. 2F).

#### NMSS (ON state)

Three studies (*n* = 502; 293 intervention, 209 control) evaluating Exenatide were included. The pooled MD was 0.72 (95% CI − 2.09 to 3.54; *p* = 0.615), indicating no statistically significant effect. No heterogeneity was observed (I² = 0.0%; τ² = 0), and the Q test was non-significant (Q = 0.10, *p* = 0.952), suggesting consistent findings across studies (Fig. 2G).

### Network meta analysis results

#### MDS-UPDRS part III (ON state)

**Fig. 3:**
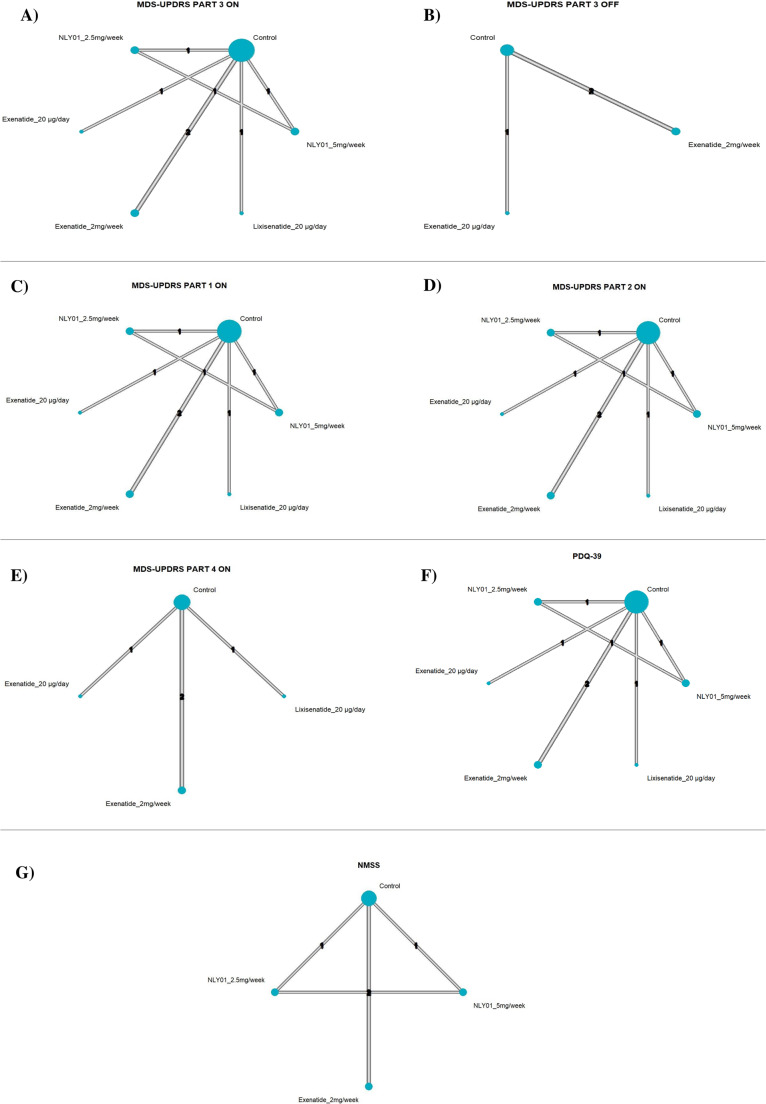
**A** Network plot of MDS-UPDRS part III (ON State). **B** Network plot of MDS-UPDRS part III (OFF State). **C** Network plot of MDS-UPDRS part I (ON State). **D** Network plot of MDS-UPDRS part II (ON State). **E** Network plot of MDS-UPDRS part IV (ON State). **F** Network plot of PDQ-39. **G** Network plot of NMSS

**Fig. 4:**
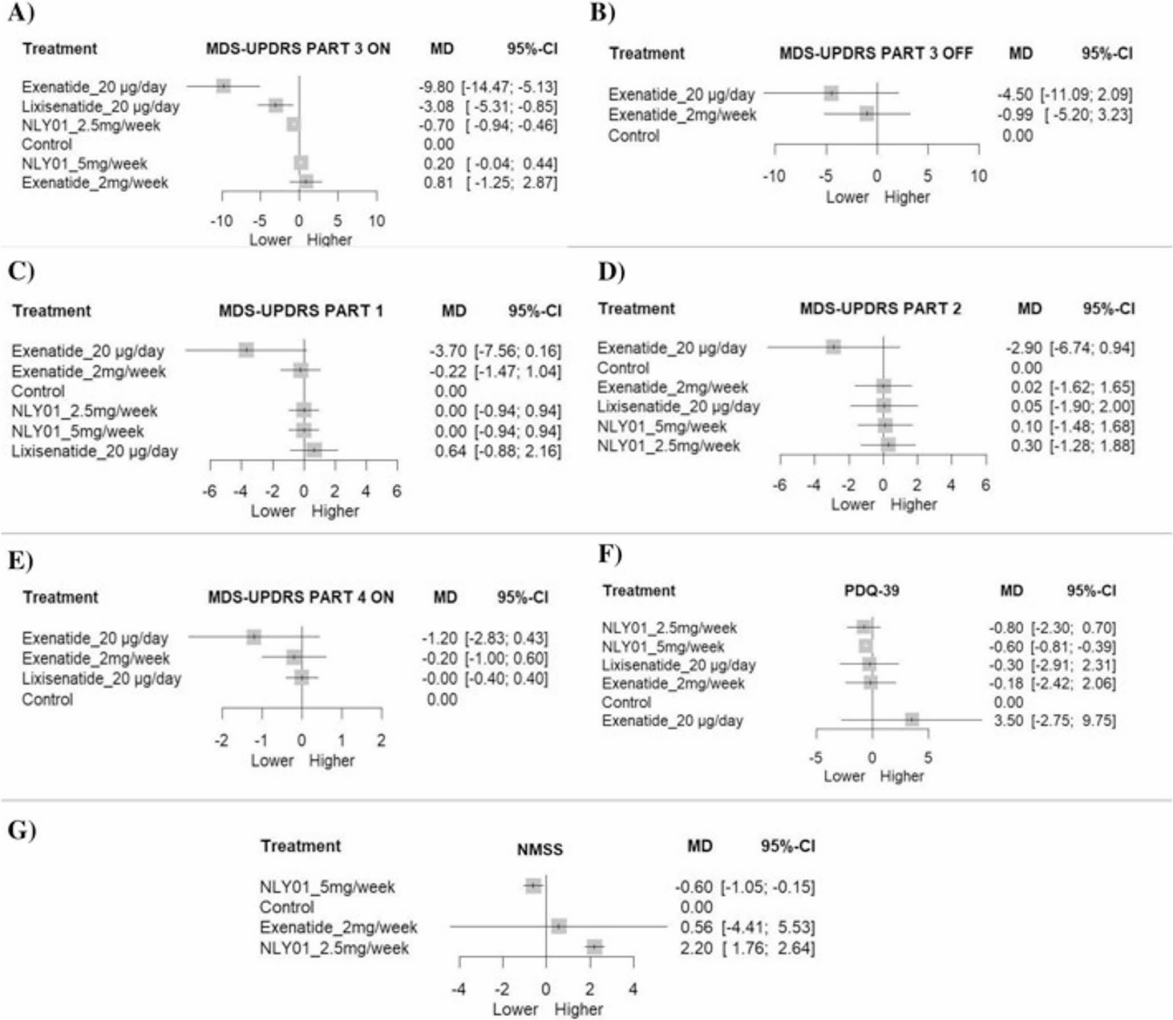
**A** Network meta analysis forest plot of MDS-UPDRS part III (ON State). **B** Network meta analysis forest plot of MDS-UPDRS part III (OFF State). **C** Network meta analysis forest plot of MDS-UPDRS part I (ON State). **D** Network meta analysis forest plot of MDS-UPDRS part II (ON State). **E** Network meta analysis forest plot of MDS-UPDRS part IV (ON State). **F** Network meta analysis forest plot of PDQ-39. **G** Network meta analysis forest plot of NMSS

Five studies (seven comparisons, six treatments) formed a connected network (Fig 3). Compared with control, Exenatide 20 µg/day produced a significant and clinically meaningful reduction (MD − 9.80; 95% CI − 14.47 to − 5.13; *p* < 0.0001). Lixisenatide 20 µg/day (MD − 3.08; 95% CI − 5.31 to − 0.85; *p* = 0.0068) and NLY01 2.5 mg/week (MD − 0.70; 95% CI − 0.94 to − 0.46; *p* < 0.0001) were also statistically significant. Exenatide 2 mg/week (MD 0.81; 95% CI − 1.25 to 2.87; *p* = 0.4413) and NLY01 5 mg/week (MD 0.20; 95% CI − 0.04 to 0.44; *p* = 0.1042) were not significant. No evidence of heterogeneity was detected (τ² = 0; I² = 0%; Q = 0.08, *p* = 0.7827) (Fig. 4A).

#### MDS-UPDRS part III (OFF state)

Three studies contributed three comparisons. Neither Exenatide 20 µg/day (MD − 4.50; 95% CI − 11.09 to 2.09; *p* = 0.1807) nor Exenatide 2 mg/week (MD − 0.99; 95% CI − 5.20 to 3.23; *p* = 0.6458) significantly differed from control. Substantial heterogeneity was observed (τ² = 6.69; I² = 72.4%), with Q approaching significance (*p* = 0.0571) (Fig. 4B).

#### MDS-UPDRS part I (ON state)

Five studies (seven comparisons) were included. Exenatide 20 µg/day showed a borderline reduction (MD − 3.70; 95% CI − 7.56 to 0.16; *p* = 0.0605), whereas Exenatide 2 mg/week was not significant (MD − 0.22; 95% CI − 1.47 to 1.04; *p* = 0.7337). Lixisenatide 20 µg/day (MD 0.64; 95% CI − 0.88 to 2.16; *p* = 0.4078) and both NLY01 doses (MD 0.00; 95% CI − 0.94 to 0.94; *p* = 1.000) showed no effect. Heterogeneity was low (τ² = 0.23; I² = 23.1%) (Fig. 4C).

#### MDS-UPDRS part II (ON state)

Across five studies, none of the treatments significantly differed from control. Exenatide 20 µg/day showed MD − 2.90 (95% CI − 6.74 to 0.94; *p* = 0.1393), Exenatide 2 mg/week MD 0.02 (95% CI − 1.62 to 1.65; *p* = 0.9834), Lixisenatide 20 µg/day MD 0.05 (95% CI − 1.90 to 2.00; *p* = 0.9599), NLY01 2.5 mg/week MD 0.30 (95% CI − 1.28 to 1.88; *p* = 0.7101), and NLY01 5 mg/week MD 0.10 (95% CI − 1.48 to 1.68; *p* = 0.9014). Moderate heterogeneity was observed (τ² = 0.65; I² = 44.8%) (Fig. 4D).

#### MDS-UPDRS part IV (ON state)

Four studies were included. No treatment showed a statistically significant difference versus control: Exenatide 20 µg/day (MD − 1.20; 95% CI − 2.83 to 0.43; *p* = 0.1503), Exenatide 2 mg/week (MD − 0.20; 95% CI − 1.00 to 0.60; *p* = 0.6254), and Lixisenatide 20 µg/day (MD 0.00; 95% CI − 0.40 to 0.40; *p* = 1.000). No heterogeneity was detected (I² = 0%) (Fig. 4E).

#### PDQ-39

Five studies were analyzed. Only NLY01 5 mg/week demonstrated a significant reduction compared with control (MD − 0.60; 95% CI − 0.81 to − 0.39; *p* < 0.0001). Exenatide 20 µg/day (MD 3.50; *p* = 0.2726), Exenatide 2 mg/week (MD − 0.18; *p* = 0.8762), Lixisenatide 20 µg/day (MD − 0.30; *p* = 0.8220), and NLY01 2.5 mg/week (MD − 0.80; *p* = 0.2945) were not significant. No heterogeneity was observed (I² = 0%) (Fig. 4F).

#### NMSS

Three studies contributed five comparisons. Exenatide 2 mg/week showed no significant effect (MD 0.56; 95% CI − 4.41 to 5.53; *p* = 0.8253). NLY01 2.5 mg/week demonstrated a significant increase (MD 2.20; 95% CI 1.76 to 2.64; *p* < 0.0001), while NLY01 5 mg/week showed a significant reduction (MD − 0.60; 95% CI − 1.05 to − 0.15; *p* = 0.0093). No evidence of heterogeneity was detected (I² = 0%) (Fig. 4G).

### Transitivity assessment

Baseline characteristics and outcome measures were compared across treatment groups using weighted linear models. No statistically significant differences were observed for baseline MDS-UPDRS Parts I–IV, PDQ-39 scores, or mean age (all overall group *p*-values > 0.10), indicating good comparability across groups. These findings support the plausibility of the transitivity assumption and the validity of indirect comparisons in the network meta-analysis (Supplementary Material).

### Safety assessment

In the random-effects meta-analysis, GLP-1 receptor agonists were associated predominantly with gastrointestinal adverse events. Nausea was significantly increased in the treatment group compared with control (RR 2.09, 95% CI 1.51–2.88; *p* < 0.0001; I² = 51.7%). Similarly, vomiting occurred more frequently among treated participants (RR 4.53, 95% CI 1.95–10.50; *p* = 0.0004; I² = 0%). Constipation was also significantly increased (RR 1.89, 95% CI 1.11–3.20; *p* = 0.019; I² = 62.2%). Diarrhoea showed a trend toward increased risk but did not reach statistical significance (RR 1.45, 95% CI 0.98–2.15; *p* = 0.061; I² = 0%). Weight loss was significantly more common in the treatment arm (RR 1.81, 95% CI 1.18–2.79; *p* = 0.0067; I² = 51.8%).

In contrast, there were no statistically significant differences between groups for fatigue (RR 1.67, 95% CI 0.74–3.78; *p* = 0.22; I² = 46.4%), headache (RR 1.20, 95% CI 0.73–1.97; *p* = 0.47; I² = 0%), anxiety (RR 1.94, 95% CI 0.48–7.80; *p* = 0.35; I² = 0%), urinary tract infection (RR 0.90, 95% CI 0.22–3.67; *p* = 0.89; I² = 52.9%), or administration site disorder (RR 0.99, 95% CI 0.84–1.16; *p* = 0.90; I² = 0%). Overall, the safety profile was characterized mainly by gastrointestinal intolerance, with low to moderate heterogeneity observed across outcomes (Supplementary Material).

### Publication bias

Assessment of publication bias was not performed because fewer than six studies were available per outcome, limiting the reliability and interpretability of funnel plots and statistical tests for small-study effects.

### Risk of bias

**Fig. 5:**
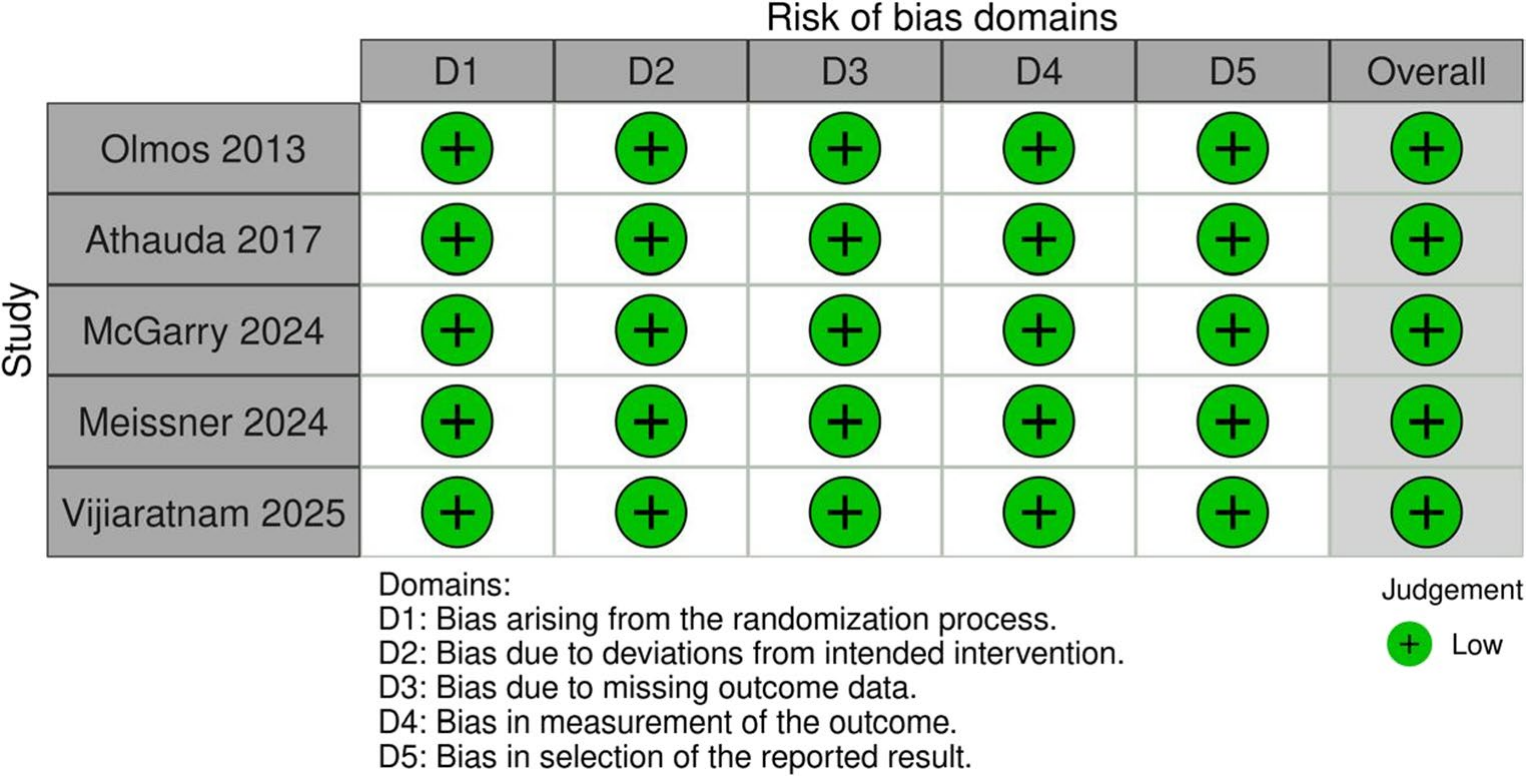
Risk of bias assessment

All five included studies were judged to have a low risk of bias across all assessed domains, indicating overall high methodological quality and a low likelihood of systematic bias influencing the pooled estimates (Fig. 5).

## Discussion

This systematic review and network meta-analysis synthesized evidence from five randomized controlled trials comprising 708 patients with Parkinson’s disease to evaluate the efficacy of GLP-1 receptor agonists across motor and non-motor domains. By integrating both pairwise and network meta-analytic approaches, we assessed a broad range of outcomes, including MDS-UPDRS Parts I–IV, PDQ-39, and NMSS, allowing both class-level and dose-specific comparisons.

Motor outcomes assessed using MDS-UPDRS Part III demonstrated the most notable signals of potential benefit, particularly in the ON state. In pairwise meta-analysis, GLP-1 receptor agonists were associated with a numerical reduction in motor scores; however, these effects did not reach statistical significance and were accompanied by substantial heterogeneity. This heterogeneity likely reflects differences in study design, dosing regimens, treatment duration, baseline disease severity, and washout protocols.

Subgroup analysis suggested a statistically significant improvement with lixisenatide; however, this finding was derived from a single study and should therefore be interpreted cautiously. Exenatide showed a consistent direction toward motor improvement across studies, but wide confidence intervals and high heterogeneity indicate variability in effect magnitude.

In contrast, network meta-analysis identified statistically significant improvements for specific regimens, notably exenatide 20 µg/day, lixisenatide 20 µg/day, and NLY01 2.5 mg/week. Importantly, no evidence of heterogeneity was observed within the ON-state network, suggesting consistent comparative estimates. These findings indicate that dose-specific effects may be more readily detected using network approaches that incorporate indirect evidence, rather than class-based aggregation alone. Overall, ON-state motor outcomes suggest a trend toward benefit, with clearer signals emerging at specific doses.

For OFF-state motor function, both pairwise and network analyses showed a consistent direction favoring GLP-1 receptor agonists; however, none of the pooled estimates reached statistical significance. Moderate to substantial heterogeneity was observed, indicating variability in treatment response across studies. The lack of significant effects in the OFF state suggests that any observed motor improvement may be more evident under optimized dopaminergic conditions, rather than reflecting a clear disease-modifying effect detectable in the medication-withdrawn state.

Across both analytical approaches, MDS-UPDRS Part I (non-motor experiences of daily living) showed no meaningful treatment effect. Effect estimates were centered near zero with low-to-moderate heterogeneity, and network analysis revealed no statistically significant benefit for any regimen. These stable and consistent findings suggest that GLP-1 receptor agonists are unlikely to substantially influence non-motor experiences within the durations studied.

Similarly, MDS-UPDRS Part II (activities of daily living) demonstrated highly consistent null results across both pairwise and network meta-analyses. Effect estimates were close to zero, confidence intervals were narrow, and heterogeneity was minimal, indicating a stable absence of detectable benefit in this domain.

MDS-UPDRS Part IV (motor complications) also showed no evidence of benefit. Both analytical approaches demonstrated effect estimates near zero with no evidence of heterogeneity (I² = 0%), suggesting that GLP-1 receptor agonists are unlikely to meaningfully affect motor complications during the studied follow-up periods.

Quality-of-life outcomes assessed using PDQ-39 were largely neutral in pairwise analysis. However, in the network meta-analysis, NLY01 5 mg/week demonstrated a statistically significant reduction in PDQ-39 scores. While this isolated finding suggests a potential dose-specific quality-of-life benefit, the absence of consistent effects across other doses or agents limits its interpretability.

NMSS outcomes were similarly neutral in pairwise analysis, with no significant effects or heterogeneity. Network analysis revealed divergent, bidirectional dose-related effects for NLY01, with one dose associated with increased scores and another with reduced scores. This instability likely reflects imprecision rather than a true therapeutic signal and underscores the uncertainty surrounding non-motor symptom outcomes.

GLP-1 receptor agonists were mainly associated with gastrointestinal adverse events, particularly nausea, vomiting, and constipation, consistent with their known mechanism of action. Weight loss was also more common, while other adverse events did not differ significantly from controls. Overall, the safety profile was largely characterized by manageable gastrointestinal intolerance.

From a biological perspective, GLP-1 receptor agonists have been hypothesized to exert neuroprotective and anti-inflammatory effects, potentially modifying disease progression in Parkinson’s disease (7). The observed ON-state motor improvements with specific regimens, particularly exenatide 20 µg/day, align with this mechanistic rationale. However, the absence of consistent benefits across OFF-state motor outcomes, non-motor symptoms, and functional domains suggests that any therapeutic effects are likely modest and context-dependent rather than broadly disease-modifying.

Several previous meta-analyses have evaluated GLP-1 receptor agonists in Parkinson’s disease; however, direct comparison with our findings should be interpreted cautiously given methodological differences and the evolving evidence base. Messak et al. (8) reported benefits in MDS-UPDRS Part III (Off) but did not include the more recent trial by Vijaratnam et al. (9). Costa et al. observed no improvements in MDS-UPDRS Part III, although their analysis was limited to placebo-controlled studies (10). Furthermore, Zhang et al. incorporated a trial by Hogg et al., which was not clearly reported as a full-text peer reviewed publication and may introduce some uncertainty (11, 12).

Strengths of this review include the inclusion of all eligible randomized controlled trials available to date and a drug- and dose-specific comparative evaluation. The methodological quality of the included studies was high, with all trials assessed as having low risk of bias using the Cochrane RoB 2.0 tool, strengthening the internal validity of our findings. Several limitations should be acknowledged. First, the evidence base is limited to five randomized trials, restricting statistical power and the robustness of indirect comparisons. Second, follow-up durations were heterogeneous, limiting conclusions regarding consistent or disease-modifying effects. Third, comparator/control varied across studies, which may introduce heterogeneity and potentially affect the assumptions underlying the network meta-analysis. Fourth, some statistically significant findings were driven by single studies or specific doses, reducing generalizability. Finally, publication bias could not be formally assessed, and small-study effects cannot be excluded.

## Conclusions

In conclusion, this systematic review and network meta-analysis suggests that GLP-1 receptor agonists may provide modest, dose-specific improvements in ON-state motor symptoms in Parkinson’s disease, particularly with higher doses of exenatide and lixisenatide. However, evidence for broader motor, non-motor, functional, or quality-of-life benefits remains inconsistent. Larger, well-powered, and longer-duration randomized trials directly comparing different GLP-1 receptor agonists and dosing strategies are needed to clarify their therapeutic role and potential disease-modifying effects in Parkinson’s disease.

## Supplementary information

Below is the link to the electronic supplementary material.

Supplementary Material 1

## Data Availability

All data generated or analyzed during this study are included in this published article and its supplementary information files. Extracted datasets and analytic code are available from the corresponding author upon reasonable request.

## Abbreviations

PD: Parkinson’s Disease
GI: Gastrointestinal
T2DM: Type 2 Diabetes Mellitus
GLP-1RAs/GLP-1 agonists: Glucagon-like Peptide-1 Receptor Agonists
CNS: Central Nervous System
ADLs: Activity of Daily Living

## Declarations

### Generative AI and AI assisted technologies in writing process

During the preparation of this work the author(s) used ChatGPT-5(OpenAI) for language refinement, summarisation, and converting statistical outputs into written text. After using this tool/service, the authors reviewed and edited the content as needed and take full responsibility for the content of the publication.
