# Supplementary material for: Efficacy of GLP-1 receptor agonists in Parkinson’s disease: a systematic review and exploratory network meta-analysis of randomized controlled trials
Source: Neurol Sci. 2026 Apr 17;47(5):432. doi: 10.1007/s10072-026-08929-1 (PMC13090280; doi:10.1007/s10072-026-08929-1)

**Supplementary appendix:**



**Index**

**Appendix 1. PRISMA checklist …………………………………………………………………………….2**

**Appendix 2. Additional details on search strategy…………………………………………………………6**

**Appendix 3. Transitivity assessments………………………………………………………………………18**

**Appendix 4. League table of effect sizes for individual medications……………………………………...24**

**Appendix 5. Treatment rankings according to P-scores…………………………………………………...29**

**Appendix 6. Safety Assessments……………………………………………………………………………..30**

**Appendix 1.**

| **Section/Topic** | **Item #** | **Checklist Item** | **Reported on Page #** |
| --- | --- | --- | --- |
| **TITLE** |  |  |  |
| Title | 1 | Identify the report as a systematic review *incorporating a network meta-analysis (or related form of meta-analysis).* | ***#1*** |
|  |  |  |  |
| **ABSTRACT** |  |  |  |
| Structured summary | 2 | Provide a structured summary including, as applicable:  **Background:** main objectives  **Methods:** data sources; study eligibility criteria, participants, and interventions; study appraisal; and *synthesis methods, such as network meta-analysis.*  **Results:** number of studies and participants identified; summary estimates with corresponding confidence/credible intervals; *treatment rankings may also be discussed. Authors may choose to summarize pairwise comparisons against a chosen treatment included in their analyses for brevity.*  **Discussion/Conclusions:** limitations; conclusions and implications of findings.  **Other:** primary source of funding; systematic review registration number with registry name. | #1 |
|  |  |  |  |
| **INTRODUCTION** |  |  |  |
| Rationale | 3 | Describe the rationale for the review in the context of what is already known*, including mention of why a network meta-analysis has been conducted.* | ***#2*** |
| Objectives | 4 | Provide an explicit statement of questions being addressed, with reference to participants, interventions, comparisons, outcomes, and study design (PICOS). |  |
|  |  |  | #2 |
| **METHODS** |  |  |  |
| Protocol and registration | 5 | Indicate whether a review protocol exists and if and where it can be accessed (e.g., Web address); and, if available, provide registration information, including registration number. | #2 |
| Eligibility criteria | 6 | Specify study characteristics (e.g., PICOS, length of follow-up) and report characteristics (e.g., years considered, language, publication status) used as criteria for eligibility, giving rationale. *Clearly describe eligible treatments included in the treatment network, and note whether any have been clustered or merged into the same node (with justification).* | ***#2*** |
| Information sources | 7 | Describe all information sources (e.g., databases with dates of coverage, contact with study authors to identify additional studies) in the search and date last searched. | #2 |
| Search | 8 | Present full electronic search strategy for at least one database, including any limits used, such that it could be repeated. | #2 |
| Study selection | 9 | State the process for selecting studies (i.e., screening, eligibility, included in systematic review, and, if applicable, included in the meta-analysis). | #2 |
| Data collection process | 10 | Describe method of data extraction from reports (e.g., piloted forms, independently, in duplicate) and any processes for obtaining and confirming data from investigators. | #2, #3 |
| Data items | 11 | List and define all variables for which data were sought (e.g., PICOS, funding sources) and any assumptions and simplifications made. | #2, #3 |
| **Geometry of the network** | **S1** | Describe methods used to explore the geometry of the treatment network under study and potential biases related to it. This should include how the evidence base has been graphically summarized for presentation, and what characteristics were compiled and used to describe the evidence base to readers. | ***#3*** |
| Risk of bias within individual studies | 12 | Describe methods used for assessing risk of bias of individual studies (including specification of whether this was done at the study or outcome level), and how this information is to be used in any data synthesis. | ***#3*** |
| Summary measures | 13 | State the principal summary measures (e.g., risk ratio, difference in means). *Also describe the use of additional summary measures assessed, such as treatment rankings and surface under the cumulative ranking curve (SUCRA) values, as well as modified approaches used to present summary findings from meta-analyses.* | ***#3-9*** |
| Planned methods of analysis | 14 | Describe the methods of handling data and combining results of studies for each network meta-analysis. This should include, but not be limited to:   - *Handling of multi-arm trials;* - *Selection of variance structure;* - *Selection of prior distributions in Bayesian analyses; and* - *Assessment of model fit.* | ***#3*** |
| **Assessment of Inconsistency** | **S2** | Describe the statistical methods used to evaluate the agreement of direct and indirect evidence in the treatment network(s) studied. Describe efforts taken to address its presence when found. | ***#Formal statistical assessment of inconsistency was not performed.*** |
| Risk of bias across studies | 15 | Specify any assessment of risk of bias that may affect the cumulative evidence (e.g., publication bias, selective reporting within studies). | **#Not performed due to the limited number of included studies.** |
| Additional analyses | 16 | Describe methods of additional analyses if done, indicating which were pre-specified. This may include, but not be limited to, the following:   - Sensitivity or subgroup analyses; - Meta-regression analyses; - *Alternative formulations of the treatment network; and* - *Use of alternative prior distributions for Bayesian analyses (if applicable).* | ***#3*** |
|  |  |  |  |
| **RESULTS†** |  |  |  |
| Study selection | 17 | Give numbers of studies screened, assessed for eligibility, and included in the review, with reasons for exclusions at each stage, ideally with a flow diagram. | #3-#4 |
| **Presentation of network structure** | **S3** | Provide a network graph of the included studies to enable visualization of the geometry of the treatment network. | #10 |
| **Summary of network geometry** | **S4** | Provide a brief overview of characteristics of the treatment network. This may include commentary on the abundance of trials and randomized patients for the different interventions and pairwise comparisons in the network, gaps of evidence in the treatment network, and potential biases reflected by the network structure. | #7-#10 |
| Study characteristics | 18 | For each study, present characteristics for which data were extracted (e.g., study size, PICOS, follow-up period) and provide the citations. | #6-#10 |
| Risk of bias within studies | 19 | Present data on risk of bias of each study and, if available, any outcome level assessment. | #9, #11 |
| Results of individual studies | 20 | For all outcomes considered (benefits or harms), present, for each study: 1) simple summary data for each intervention group, and 2) effect estimates and confidence intervals. *Modified approaches may be needed to deal with information from larger networks.* | #Individual study data are presented in Table 1 and corresponding forest plots, from which effect estimates for each study can be obtained. |
| Synthesis of results | 21 | Present results of each meta-analysis done, including confidence/credible intervals. *In larger networks, authors may focus on comparisons versus a particular comparator (e.g. placebo or standard care), with full findings presented in an appendix. League tables and forest plots may be considered to summarize pairwise comparisons.* If additional summary measures were explored (such as treatment rankings), these should also be presented. | #3-#10, appendix |
| **Exploration for inconsistency** | **S5** | Describe results from investigations of inconsistency. This may include such information as measures of model fit to compare consistency and inconsistency models, *P* values from statistical tests, or summary of inconsistency estimates from different parts of the treatment network. | #Formal statistical assessment of inconsistency was not performed; however, transitivity assumptions were evaluated and are reported in the appendix. |
| Risk of bias across studies | 22 | Present results of any assessment of risk of bias across studies for the evidence base being studied. | #Not performed due to the limited number of included studies. |
| Results of additional analyses | 23 | Give results of additional analyses, if done (e.g., sensitivity or subgroup analyses, meta-regression analyses*, alternative network geometries studied, alternative choice of prior distributions for Bayesian analyses,* and so forth). | #8-#9 |
|  |  |  |  |
| **DISCUSSION** |  |  |  |
| Summary of evidence | 24 | Summarize the main findings, including the strength of evidence for each main outcome; consider their relevance to key groups (e.g., healthcare providers, users, and policy-makers). | #9-#11 |
| Limitations | 25 | Discuss limitations at study and outcome level (e.g., risk of bias), and at review level (e.g., incomplete retrieval of identified research, reporting bias). *Comment on the validity of the assumptions, such as transitivity and consistency. Comment on any concerns regarding network geometry (e.g., avoidance of certain comparisons).* | #11 |
| Conclusions | 26 | Provide a general interpretation of the results in the context of other evidence, and implications for future research. | #11 |
|  |  |  |  |
| **FUNDING** |  |  | #12 |
| Funding | 27 | Describe sources of funding for the systematic review and other support (e.g., supply of data); role of funders for the systematic review. This should also include information regarding whether funding has been received from manufacturers of treatments in the network and/or whether some of the authors are content experts with professional conflicts of interest that could affect use of treatments in the network. |  |

PICOS = population, intervention, comparators, outcomes, study design.

* Text in italics indicates wording specific to reporting of network meta-analyses that has been added to guidance from the PRISMA statement.

† Authors may wish to plan for use of appendices to present all relevant information in full detail for items in this section.

**Appendix 2.**

**Supplementary Table S1**

**The adjusted search terms as per searched electronic databases [as of 10.02.2026]**

| Database | No | Search Query | Results |
| --- | --- | --- | --- |
| Scopus | | | |
|  | ( TITLE-ABS-KEY ( ( "Parkinsonian Disorders" OR "Parkinsonism" OR "Parkinsonian Syndrome" OR "Parkinsonian Diseases" OR "Parkinsonian Syndromes" OR "Autosomal Dominant Juvenile Parkinson Disease" OR "Juvenile Parkinsonism, Autosomal Dominant" OR "Autosomal Dominant Juvenile Parkinsonism" OR "Juvenile Parkinson Disease, Autosomal Dominant" OR "Parkinson Disease, Autosomal Dominant Juvenile" OR "Parkinson Disease, Juvenile, Autosomal Dominant" OR "Parkinsonism, Juvenile, Autosomal Dominant" OR "Parkinsonism, Experimental" OR "Experimental Parkinsonisms" OR "Parkinsonisms, Experimental" OR "Experimental Parkinson Disease" OR "Diseases, Experimental Parkinson" OR "Experimental Parkinson Diseases" OR "Parkinson Diseases, Experimental" OR "Experimental Parkinsonism" OR "Experimental Parkinsonism, MPTP-Induced" OR "Experimental Parkinsonism, MPTP Induced" OR "Parkinsonism, MPTP-Induced Experimental" OR "MPTP-Induced Experimental Parkinsonism" OR "MPTP Induced Experimental Parkinsonism" OR "Parkinson Disease, Experimental" OR "Parkinsonism, Juvenile" OR "Juvenile Parkinsonism" OR "Juvenile Parkinsonisms" OR "Parkinsonisms, Juvenile" OR "Juvenile Parkinson Disease" OR "Parkinson Disease, Juvenile" OR "Autosomal Recessive Juvenile Parkinson Disease" OR "Chromosome 6-Linked Autosomal Recessive Parkinsonism" OR "Chromosome 6 Linked Autosomal Recessive Parkinsonism" OR "Juvenile Parkinson Disease, Autosomal Recessive" OR "Juvenile Parkinsonism, Autosomal Recessive" OR "Parkinsonism, Juvenile, Autosomal Recessive" OR "Familial Parkinson Disease, Autosomal Recessive" OR "Parkinson Disease, Familial, Autosomal Recessive" OR "Parkinson Disease 2, Autosomal Recessive Juvenile" OR "Parkinsonism, Early-Onset, With Diurnal Fluctuation" OR "Parkinson Disease, Juvenile, Autosomal Recessive" OR "Parkinson Disease 2" OR "Parkinson Disease Autosomal Recessive, Early Onset" OR "Parkinsonism, Early Onset, with Diurnal Fluctuation" OR "Autosomal Recessive Juvenile Parkinsonism" OR "Familial Juvenile Parkinsonism" OR "Juvenile Parkinsonism, Familial" OR "Parkinsonism, Familial Juvenile" OR "Autosomal Dominant Parkinsonism" OR "Dominant Parkinsonism, Autosomal" OR "Parkinsonism, Autosomal Dominant" OR "Ramsay Hunt Paralysis Syndrome" OR "Autosomal Recessive Parkinsonism" OR "Parkinsonism, Autosomal Recessive" OR "Recessive Parkinsonism, Autosomal" ) ) AND TITLE-ABS-KEY ( ('glucagon like peptide 1 receptor agonists'/exp OR 'glucagon like peptide 1 receptor agonists' OR 'glp-1 agonists' OR 'agonists, glp-1' OR 'glp 1 agonists' OR 'glp-1 receptor agonists' OR 'glp 1 receptor agonists' OR 'glp1 agonists' OR 'agonists, glp1' OR 'glp-1r agonists' OR 'agonists, glp-1r' OR 'glp 1r agonists' OR 'glp1r agonists' OR 'agonists, glp1r' OR 'incretin mimetics'/exp OR 'incretin mimetics' OR 'glp-1 analogs' OR 'glp 1 analogs' OR 'lixisenatide'/exp OR lixisenatide OR 'exenatide'/exp OR exenatide OR 'nly01'/exp OR nly01 OR 'glucagon-like peptide 1'/exp OR 'glucagon-like peptide 1' OR 'glucagon-like peptide-1'/exp OR 'glucagon-like peptide-1' OR glp1 OR 'glp 1'/exp OR 'glp 1' OR 'glucagon-like peptide-1 receptor agonists'/exp OR 'glucagon-like peptide-1 receptor agonists' ) ) AND TITLE-ABS-KEY ( ( "clinical trials" OR "clinical trials as a topic" OR "randomized controlled trial" OR "Randomized Controlled Trials as Topic" OR "controlled clinical trial" OR "Controlled Clinical Trials" OR "random allocation" OR "Double-Blind Method" OR "Single-Blind Method" OR "Cross-Over Studies" OR "Placebos" OR "multicenter study" OR "double blind procedure" OR "single blind procedure" OR "crossover procedure" OR "clinical trial" OR "controlled study" OR "randomization" OR "placebo" OR "randomly allocated" OR "allocated randomly" OR "factorial design" OR "factorial trial" OR "blind*" OR "rct*" OR "random*" ) ) AND TITLE-ABS-KEY ( treat* OR manag* ) ) |  | 65 |
| Web Of Science | | | |
|  | "Parkinsonian Disorders" OR "Parkinsonism" OR "Parkinsonian Syndrome" OR "Autosomal Dominant Juvenile Parkinson Disease" OR "Autosomal Recessive Juvenile Parkinsonism" OR "Juvenile Parkinsonism" OR "Experimental Parkinsonism" OR "MPTP-Induced Experimental Parkinsonism" OR "Parkinson Disease, Juvenile” (All Fields) and "Glucagon Like Peptide 1 Receptor Agonists" OR "GLP-1 Agonists" OR "Agonists, GLP-1" OR "GLP 1 Agonists" OR "GLP-1 Receptor Agonists" OR "GLP 1 Receptor Agonists" OR "GLP1 Agonists" OR "Agonists, GLP1" OR "GLP-1R Agonists" OR "Agonists, GLP-1R" OR "GLP 1R Agonists" OR "GLP1R Agonists" OR "Agonists, GLP1R" OR "Incretin Mimetics" OR "GLP-1 Analogs" OR "GLP 1 Analogs" OR lixisenatide OR exenatide OR NLY01 OR "glucagon-like peptide 1" OR "glucagon-like peptide-1" OR GLP1 OR GLP-1 OR "GLP 1" OR "Glucagon-Like Peptide-1 Receptor Agonists" (All Fields) and "clinical trials" OR "clinical trials as a topic" OR "randomized controlled trial" OR "Randomized Controlled Trials as Topic" OR "controlled clinical trial" OR "Controlled Clinical Trials" OR "random allocation" OR "Double-Blind Method" OR "Single-Blind Method" OR "Cross-Over Studies" OR "Placebos" OR "multicenter study" OR "double blind procedure" OR "single blind procedure" OR "crossover procedure" OR "clinical trial" OR "controlled study" OR "randomization" OR "placebo" OR "randomly allocated" OR "allocated randomly" OR "factorial design" OR "factorial trial" OR "blind*" OR "rct*" OR "random*" (All Fields) and 2015 or 2016 or 2017 or 2018 or 2019 or 2021 or 2022 or 2023 (Publication Years) and Article (Document Types) |  | 903 |
| EMBASE | | | |
| 1. | 'glucagon like peptide 1 receptor agonists'/exp OR 'glucagon like peptide 1 receptor agonists' OR 'glp-1 agonists' OR 'agonists, glp-1' OR 'glp 1 agonists' OR 'glp-1 receptor agonists' OR 'glp 1 receptor agonists' OR 'glp1 agonists' OR 'agonists, glp1' OR 'glp-1r agonists' OR 'agonists, glp-1r' OR 'glp 1r agonists' OR 'glp1r agonists' OR 'agonists, glp1r' OR 'incretin mimetics'/exp OR 'incretin mimetics' OR 'glp-1 analogs' OR 'glp 1 analogs' OR 'lixisenatide'/exp OR lixisenatide OR 'exenatide'/exp OR exenatide OR 'nly01'/exp OR nly01 OR 'glucagon-like peptide 1'/exp OR 'glucagon-like peptide 1' OR 'glucagon-like peptide-1'/exp OR 'glucagon-like peptide-1' OR glp1 OR 'glp 1'/exp OR 'glp 1' OR 'glucagon-like peptide-1 receptor agonists'/exp OR 'glucagon-like peptide-1 receptor agonists' |  | 80,193 |
| #2 | 'parkinsonian disorders'/exp OR 'parkinsonian disorders' OR 'parkinsonism'/exp OR 'parkinsonism' OR 'parkinsonian syndrome'/exp OR 'parkinsonian syndrome' OR 'parkinsonian diseases' OR 'parkinsonian syndromes' OR 'autosomal dominant juvenile parkinson disease' OR 'juvenile parkinsonism, autosomal dominant' OR 'autosomal dominant juvenile parkinsonism' OR 'juvenile parkinson disease, autosomal dominant' OR 'parkinson disease, autosomal dominant juvenile' OR 'parkinson disease, juvenile, autosomal dominant' OR 'parkinsonism, juvenile, autosomal dominant' OR 'parkinsonism, experimental' OR 'experimental parkinsonisms' OR 'parkinsonisms, experimental' OR 'experimental parkinson disease'/exp OR 'experimental parkinson disease' OR 'diseases, experimental parkinson' OR 'experimental parkinson diseases' OR 'parkinson diseases, experimental' OR 'experimental parkinsonism'/exp OR 'experimental parkinsonism' OR 'experimental parkinsonism, mptp-induced' OR 'experimental parkinsonism, mptp induced' OR 'parkinsonism, mptp-induced experimental' OR 'mptp-induced experimental parkinsonism' OR 'mptp induced experimental parkinsonism' OR 'parkinson disease, experimental' OR 'parkinsonism, juvenile' OR 'juvenile parkinsonism'/exp OR 'juvenile parkinsonism' OR 'juvenile parkinsonisms' OR 'parkinsonisms, juvenile' OR 'juvenile parkinson disease'/exp OR 'juvenile parkinson disease' OR 'parkinson disease, juvenile' OR 'autosomal recessive juvenile parkinson disease' OR 'chromosome 6-linked autosomal recessive parkinsonism' OR 'chromosome 6 linked autosomal recessive parkinsonism' OR 'juvenile parkinson disease, autosomal recessive' OR 'juvenile parkinsonism, autosomal recessive' OR 'parkinsonism, juvenile, autosomal recessive' OR 'familial parkinson disease, autosomal recessive' OR 'parkinson disease, familial, autosomal recessive' OR 'parkinson disease 2, autosomal recessive juvenile' OR 'parkinsonism, early-onset, with diurnal fluctuation' OR 'parkinson disease, juvenile, autosomal recessive' OR 'parkinson disease 2' OR 'parkinson disease autosomal recessive, early onset' OR 'parkinsonism, early onset, with diurnal fluctuation' OR 'autosomal recessive juvenile parkinsonism'/exp OR 'autosomal recessive juvenile parkinsonism' OR 'familial juvenile parkinsonism' OR 'juvenile parkinsonism, familial' OR 'parkinsonism, familial juvenile' OR 'autosomal dominant parkinsonism' OR 'dominant parkinsonism, autosomal' OR 'parkinsonism, autosomal dominant' OR 'ramsay hunt paralysis syndrome' OR 'autosomal recessive parkinsonism' OR 'parkinsonism, autosomal recessive' OR 'recessive parkinsonism, autosomal' |  | 58,997 |
| #3 | #1 AND #2 |  | 5,473 |
| #4 | randomized AND controlled AND trial |  | 1,215,616 |
| #5 | controlled AND clinical AND trial |  | 555,131 |
| #6 | randomized |  | 1,520,638 |
| #7 | placebo |  | 555,131 |
| #8 | clinical AND trials |  | 945,436 |
| #9 | randomly |  | 589,048 |
| #10 | trial |  | 2,786,212 |
| #11 | #4 OR #5 OR #6 OR #7 OR #8 OR #9 OR #10 |  | 3,781,397 |
| #12 | animals NOT humans |  | 940,344 |
| #13 | #11 NOT #12 |  | 3,698,560 |
| #14 | treat* OR manag* |  | 13,643,235 |
| #15 | #3 AND #13 AND #14 |  | 100 |
| PubMed | | | |
| #1 | ("Glucagon Like Peptide 1 Receptor Agonists" OR "GLP-1 Agonists" OR "Agonists, GLP-1" OR "GLP 1 Agonists" OR "GLP-1 Receptor Agonists" OR "GLP 1 Receptor Agonists" OR "GLP1 Agonists" OR "Agonists, GLP1" OR "GLP-1R Agonists" OR "Agonists, GLP-1R" OR "GLP 1R Agonists" OR "GLP1R Agonists" OR "Agonists, GLP1R" OR "Incretin Mimetics" OR "GLP-1 Analogs" OR "GLP 1 Analogs" OR lixisenatide OR exenatide OR NLY01 OR "glucagon-like peptide 1" OR "glucagon-like peptide-1" OR GLP1 OR GLP-1 OR "GLP 1" OR "Glucagon-Like Peptide-1 Receptor Agonists") |  | 33,757 |
| #2 | “Parkinsonian Disorders" OR "Parkinsonism" OR "Parkinsonian Syndrome" OR "Parkinsonian Diseases" OR "Parkinsonian Syndromes" OR "Autosomal Dominant Juvenile Parkinson Disease" OR "Juvenile Parkinsonism, Autosomal Dominant" OR "Autosomal Dominant Juvenile Parkinsonism" OR "Juvenile Parkinson Disease, Autosomal Dominant" OR "Parkinson Disease, Autosomal Dominant Juvenile" OR "Parkinson Disease, Juvenile, Autosomal Dominant" OR "Parkinsonism, Juvenile, Autosomal Dominant" OR "Parkinsonism, Experimental" OR "Experimental Parkinsonisms" OR "Parkinsonisms, Experimental" OR "Experimental Parkinson Disease" OR "Diseases, Experimental Parkinson" OR "Experimental Parkinson Diseases" OR "Parkinson Diseases, Experimental" OR "Experimental Parkinsonism" OR "Experimental Parkinsonism, MPTP-Induced" OR "Experimental Parkinsonism, MPTP Induced" OR "Parkinsonism, MPTP-Induced Experimental" OR "MPTP-Induced Experimental Parkinsonism" OR "MPTP Induced Experimental Parkinsonism" OR "Parkinson Disease, Experimental" OR "Parkinsonism, Juvenile" OR "Juvenile Parkinsonism" OR "Juvenile Parkinsonisms" OR "Parkinsonisms, Juvenile" OR "Juvenile Parkinson Disease" OR "Parkinson Disease, Juvenile" OR "Autosomal Recessive Juvenile Parkinson Disease" OR "Chromosome 6-Linked Autosomal Recessive Parkinsonism" OR "Chromosome 6 Linked Autosomal Recessive Parkinsonism" OR "Juvenile Parkinson Disease, Autosomal Recessive" OR "Juvenile Parkinsonism, Autosomal Recessive" OR "Parkinsonism, Juvenile, Autosomal Recessive" OR "Familial Parkinson Disease, Autosomal Recessive" OR "Parkinson Disease, Familial, Autosomal Recessive" OR "Parkinson Disease 2, Autosomal Recessive Juvenile" OR "Parkinsonism, Early-Onset, With Diurnal Fluctuation" OR "Parkinson Disease, Juvenile, Autosomal Recessive" OR "Parkinson Disease 2" OR "Parkinson Disease Autosomal Recessive, Early Onset" OR "Parkinsonism, Early Onset, with Diurnal Fluctuation" OR "Autosomal Recessive Juvenile Parkinsonism" OR "Familial Juvenile Parkinsonism" OR "Juvenile Parkinsonism, Familial" OR "Parkinsonism, Familial Juvenile" OR "Autosomal Dominant Parkinsonism" OR "Dominant Parkinsonism, Autosomal" OR "Parkinsonism, Autosomal Dominant" OR "Ramsay Hunt Paralysis Syndrome" OR "Autosomal Recessive Parkinsonism" OR "Parkinsonism, Autosomal Recessive" OR "Recessive Parkinsonism, Autosomal") |  | 117,242 |
| #3 | #1 AND #2 |  | 3,418 |
| #4 | randomized controlled trial [pt] |  | 624,084 |
| #5 | controlled clinical trial [pt] |  | 714,786 |
| #6 | randomized [tiab] |  | 725,925 |
| #7 | placebo [tiab] |  | 259,632 |
| #8 | clinical trials as topic [mesh:noexp] |  | 203,486 |
| #9 | randomly [tiab] |  | 444,289 |
| #10 | trial [ti] |  | 319,109 |
| #11 | #4 OR #5 OR #6 OR #7 OR #8 OR #9 OR #10 |  | 1,646,553 |
| #12 | animals [mh] NOT humans [mh] |  | 5,264,283 |
| #13 | #11 NOT #12 |  | 1,518,155 |
| #14 | Treat* OR manag* |  | 9,334,239 |
| #15 | #3 AND #13 AND #14 |  | 50 |

**Appendix 3.**

**Figure S1:** Baseline Mean Age Across Treatment Nodes (Transitivity Assessment)


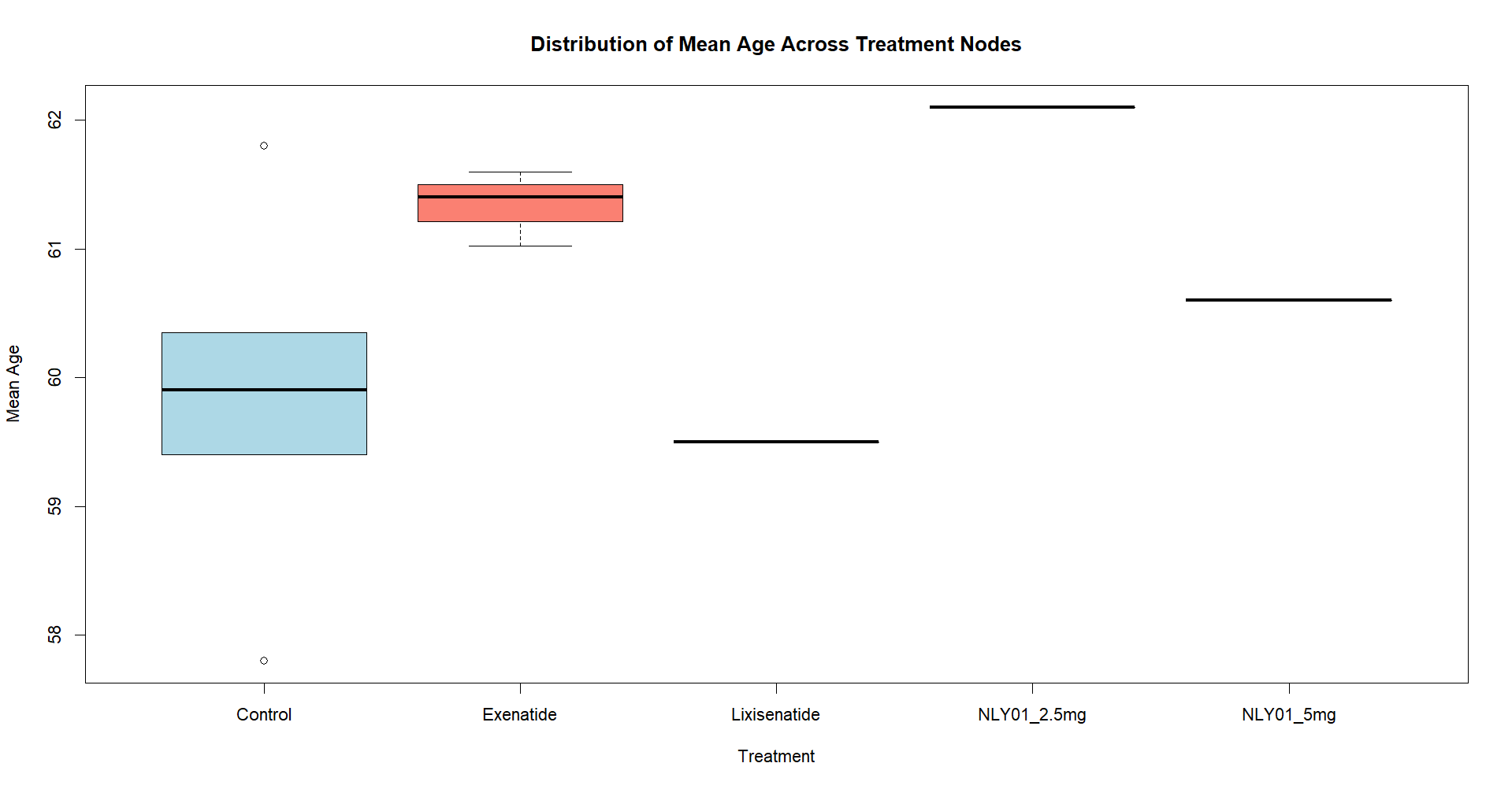


**Figure S2:** Baseline % Male Across Treatment Nodes (Transitivity Assessment)


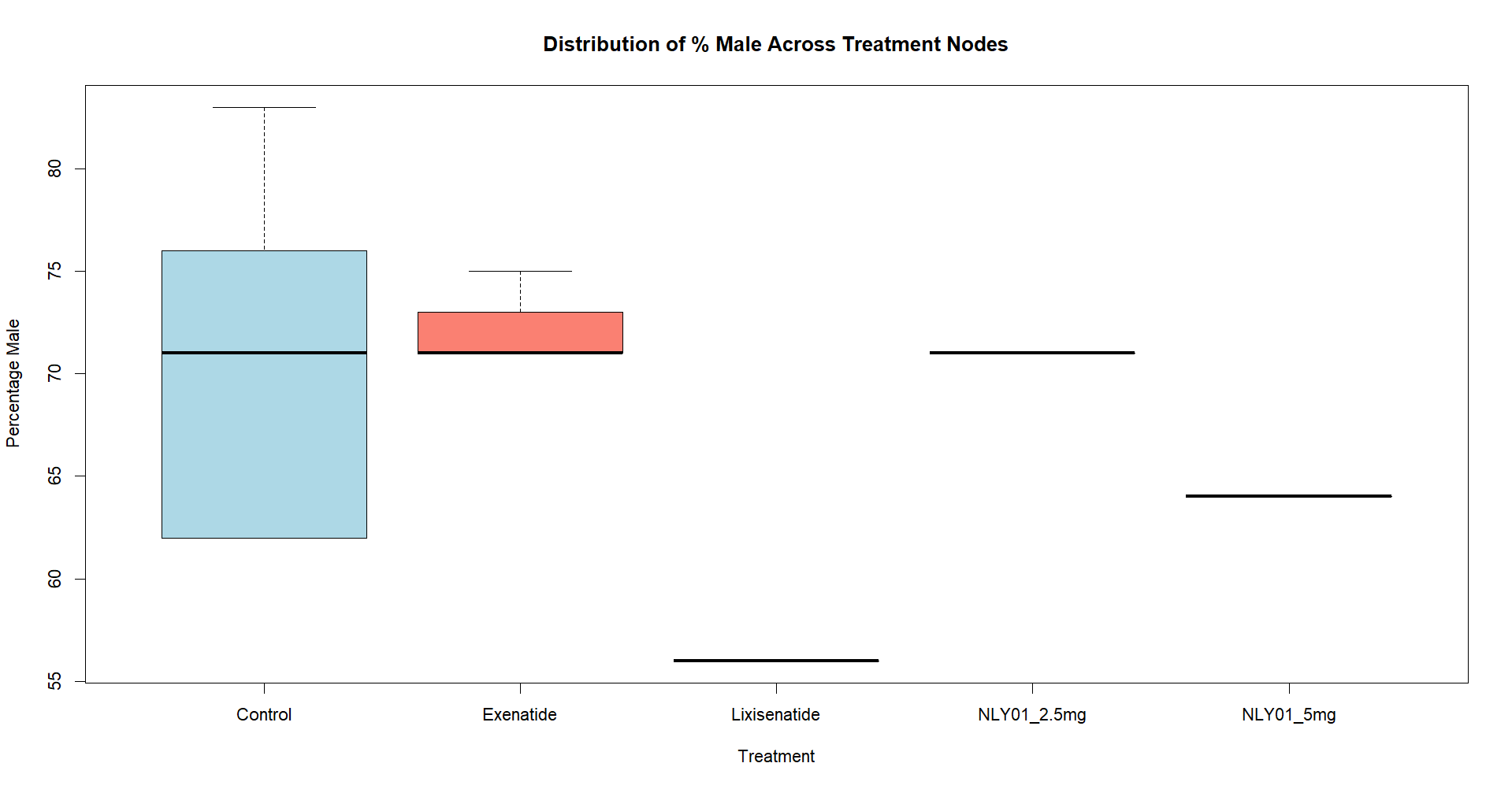


**Figure S3:** Baseline MDS UPDRS PART-1 ON Across Treatment Nodes (Transitivity Assessment)


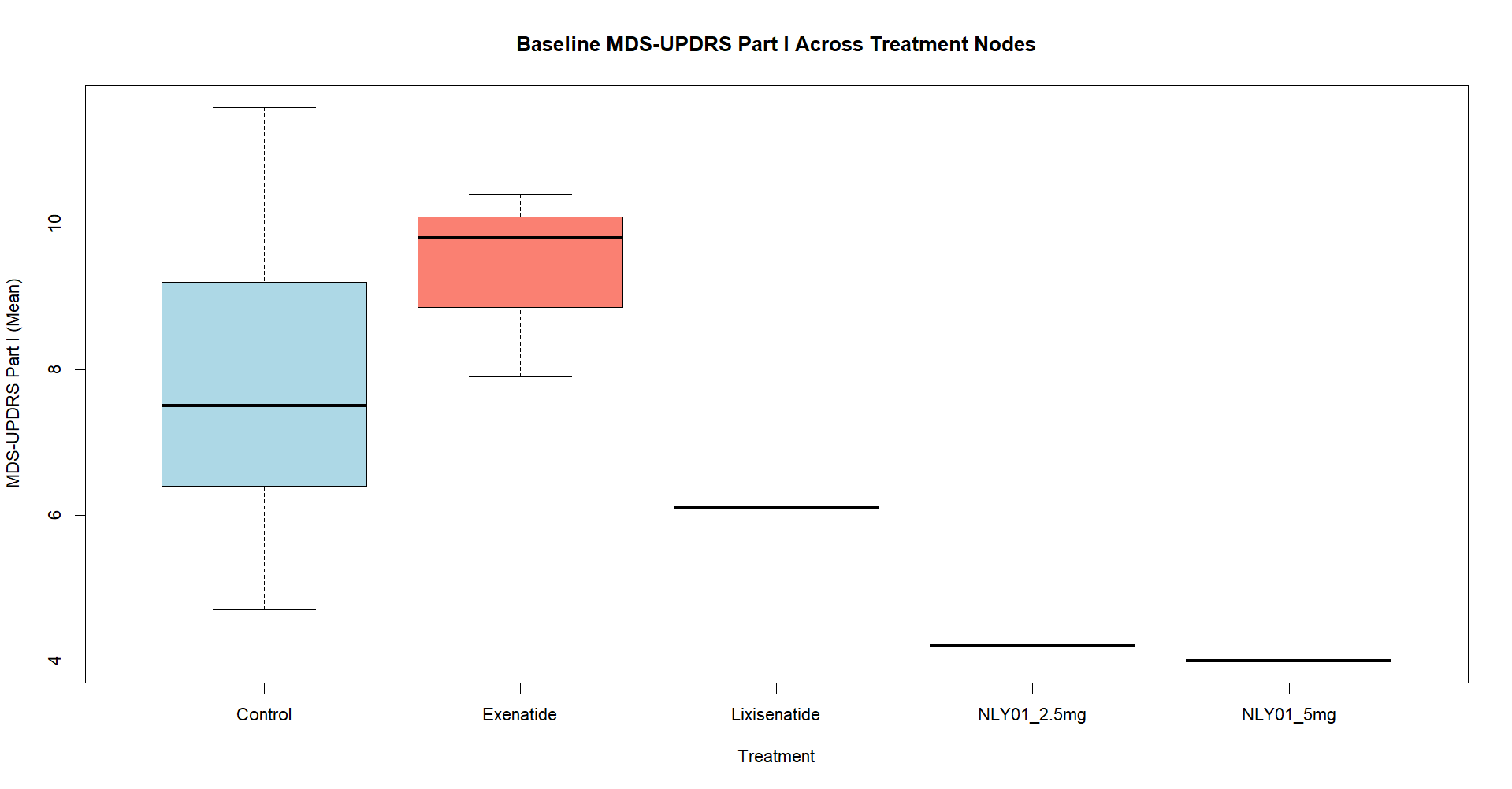


**Figure S4:** Baseline MDS UPDRS PART II ON Treatment Nodes (Transitivity Assessment)


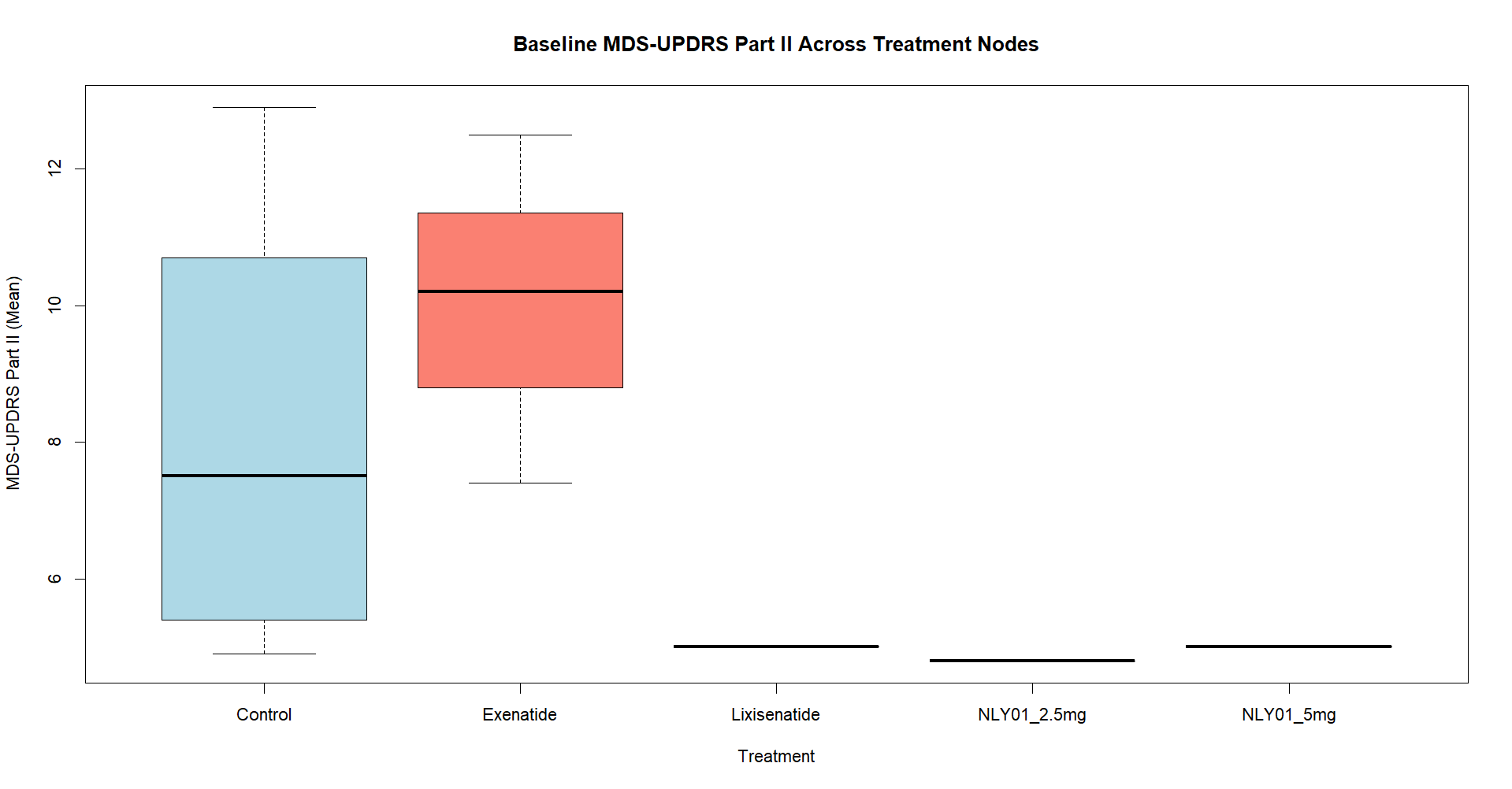


**Figure S5:** Baseline MDS UPDRS PART III ON Across Treatment Nodes (Transitivity Assessment)


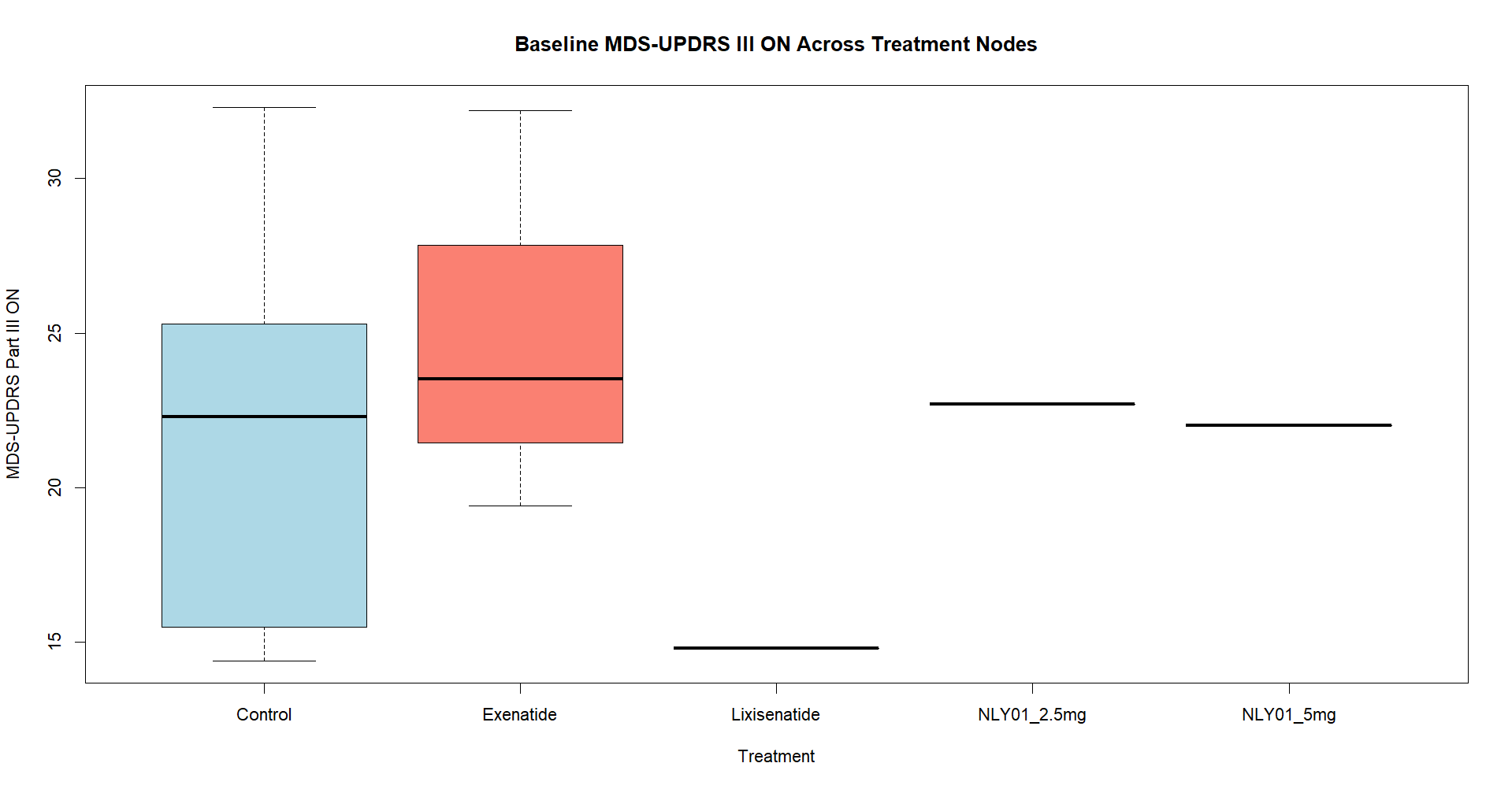


**Figure S6:** Baseline MDS UPDRS PART 4 ON Across Treatment Nodes (Transitivity Assessment)


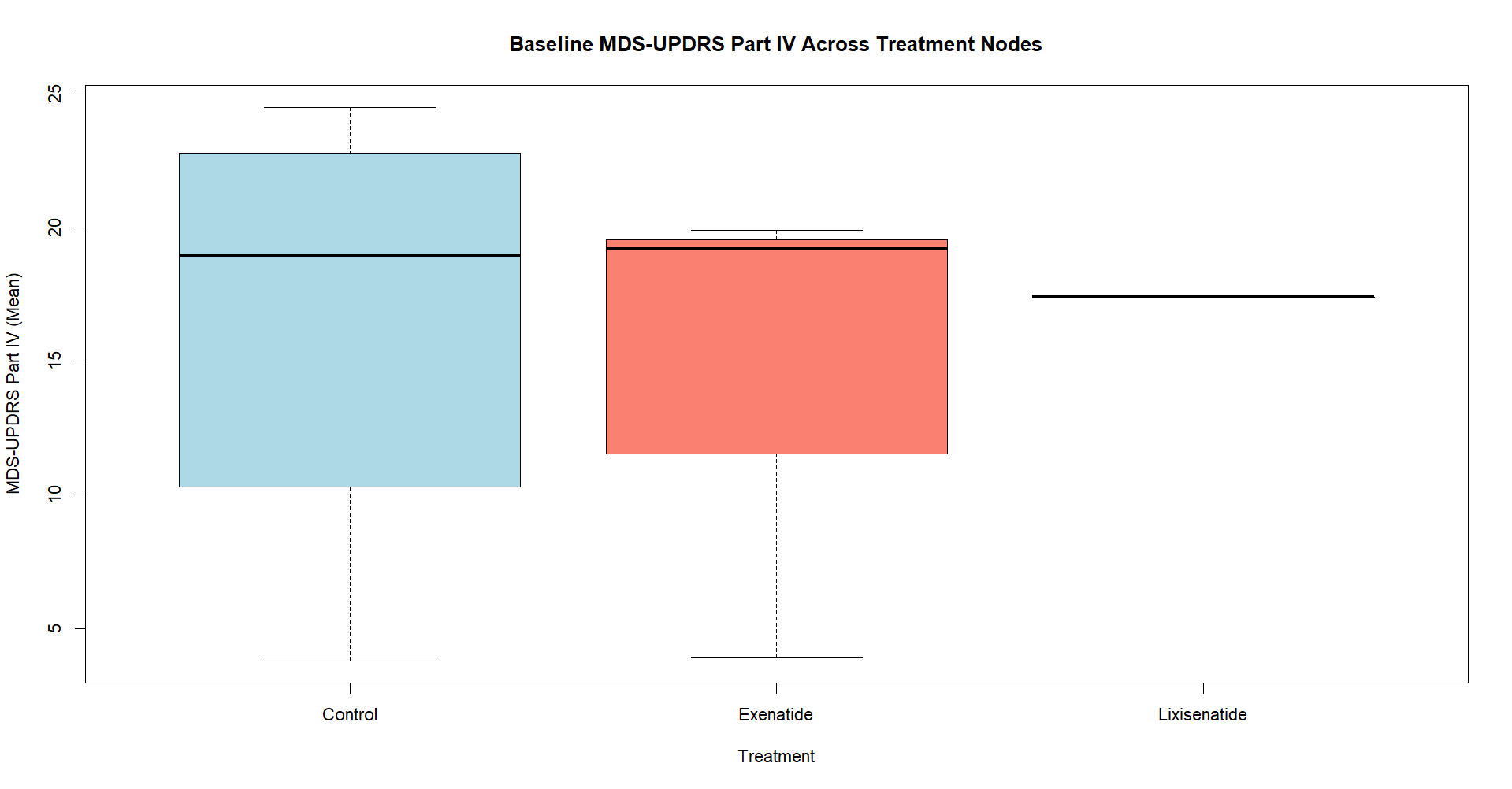


**Appendix 4.**

**Supplementary Table S2. League Table for MDS UPDRS Part 3 ON**

| **Control** | **Exenatide_20 µg/day** | **Exenatide_2mg/week** | **Lixisenatide_20 µg/day** | **NLY01_2.5mg/week** | **NLY01_5mg/week** |
| --- | --- | --- | --- | --- | --- |
| **Control** | -9.80 (-14.47 to -5.13) | 0.81 ( -1.25 to 2.87) | -3.08 ( -5.31 to -0.85) | -0.70 ( -0.94 to -0.46) | 0.20 ( -0.04 to 0.44) |
| 9.80 ( 5.13 to 14.47) | **Exenatide_20 µg/day** | 10.61 ( 5.51 to 15.71) | 6.72 ( 1.55 to 11.89) | 9.10 ( 4.42 to 13.78) | 10.00 ( 5.32 to 14.68) |
| -0.81 ( -2.87 to 1.25) | -10.61 (-15.71 to -5.51) | **Exenatide_2mg/week** | -3.89 ( -6.93 to -0.85) | -1.51 ( -3.59 to 0.57) | -0.61 ( -2.69 to 1.47) |
| 3.08 ( 0.85 to 5.31) | -6.72 (-11.89 to -1.55) | 3.89 ( 0.85 to 6.93) | **Lixisenatide_20 µg/day** | 2.38 ( 0.14 to 4.62) | 3.28 ( 1.04 to 5.52) |
| 0.70 ( 0.46 to 0.94) | -9.10 (-13.78 to -4.42) | 1.51 ( -0.57 to 3.59) | -2.38 ( -4.62 to -0.14) | **NLY01_2.5mg/week** | 0.90 ( 0.66 to 1.14) |
| -0.20 ( -0.44 to 0.04) | -10.00 (-14.68 to -5.32) | 0.61 ( -1.47 to 2.69) | -3.28 ( -5.52 to -1.04) | -0.90 ( -1.14 to -0.66) | **NLY01_5mg/week** |

**Supplementary Table S3. League Table for MDS UPDRS Part 3 OFF**

| **Control** | **Exenatide_20 µg/day** | **Exenatide_2mg/week** |
| --- | --- | --- |
| **Control** | -4.50 (-11.09 to 2.09) | -0.99 ( -5.20 to 3.23) |
| 4.50 ( -2.09 to 11.09) | **Exenatide_20 µg/day** | 3.51 ( -4.31 to 11.33) |
| 0.99 ( -3.23 to 5.20) | -3.51 (-11.33 to 4.31) | **Exenatide_2mg/week** |

**Supplementary Table S4. League Table for MDS UPDRS Part 1 ON**

| **Control** | **Exenatide_20 µg/day** | **Exenatide_2mg/week** | **Lixisenatide_20 µg/day** | **NLY01_2.5mg/week** | **NLY01_5mg/week** |
| --- | --- | --- | --- | --- | --- |
| **Control** | -3.70 (-7.56 to 0.16) | -0.22 (-1.47 to 1.04) | 0.64 (-0.88 to 2.16) | 0.00 (-0.94 to 0.94) | 0.00 (-0.94 to 0.94) |
| 3.70 (-0.16 to 7.56) | **Exenatide_20 µg/day** | 3.48 (-0.58 to 7.54) | 4.34 ( 0.19 to 8.49) | 3.70 (-0.28 to 7.68) | 3.70 (-0.28 to 7.68) |
| 0.22 (-1.04 to 1.47) | -3.48 (-7.54 to 0.58) | **Exenatide_2mg/week** | 0.86 (-1.11 to 2.83) | 0.22 (-1.35 to 1.79) | 0.22 (-1.35 to 1.79) |
| -0.64 (-2.16 to 0.88) | -4.34 (-8.49 to -0.19) | -0.86 (-2.83 to 1.11) | **Lixisenatide_20 µg/day** | -0.64 (-2.42 to 1.14) | -0.64 (-2.42 to 1.14) |
| -0.00 (-0.94 to 0.94) | -3.70 (-7.68 to 0.28) | -0.22 (-1.79 to 1.35) | 0.64 (-1.14 to 2.42) | **NLY01_2.5mg/week** | 0.00 (-0.94 to 0.94) |
| -0.00 (-0.94 to 0.94) | -3.70 (-7.68 to 0.28) | -0.22 (-1.79 to 1.35) | 0.64 (-1.14 to 2.42) | -0.00 (-0.94 to 0.94) | **NLY01_5mg/week** |

**Supplementary Table S5. League Table for MDS UPDRS Part 2 ON**

| **Control** | **Exenatide_20 µg/day** | **Exenatide_2mg/week** | **Lixisenatide_20 µg/day** | **NLY01_2.5mg/week** | **NLY01_5mg/week** |
| --- | --- | --- | --- | --- | --- |
| **Control** | -2.90 (-6.74 to 0.94) | 0.02 (-1.62 to 1.65) | 0.05 (-1.90 to 2.00) | 0.30 (-1.28 to 1.88) | 0.10 (-1.48 to 1.68) |
| 2.90 (-0.94 to 6.74) | **Exenatide_20 µg/day** | 2.92 (-1.26 to 7.09) | 2.95 (-1.36 to 7.26) | 3.20 (-0.96 to 7.36) | 3.00 (-1.16 to 7.16) |
| -0.02 (-1.65 to 1.62) | -2.92 (-7.09 to 1.26) | **Exenatide_2mg/week** | 0.03 (-2.51 to 2.58) | 0.28 (-1.99 to 2.56) | 0.08 (-2.19 to 2.36) |
| -0.05 (-2.00 to 1.90) | -2.95 (-7.26 to 1.36) | -0.03 (-2.58 to 2.51) | **Lixisenatide_20 µg/day** | 0.25 (-2.26 to 2.76) | 0.05 (-2.46 to 2.56) |
| -0.30 (-1.88 to 1.28) | -3.20 (-7.36 to 0.96) | -0.28 (-2.56 to 1.99) | -0.25 (-2.76 to 2.26) | **NLY01_2.5mg/week** | -0.20 (-1.78 to 1.38) |
| -0.10 (-1.68 to 1.48) | -3.00 (-7.16 to 1.16) | -0.08 (-2.36 to 2.19) | -0.05 (-2.56 to 2.46) | 0.20 (-1.38 to 1.78) | **NLY01_5mg/week** |

**Supplementary Table S6. League Table for MDS UPDRS Part 4 ON**

| **Control** | **Exenatide_20 µg/day** | **Exenatide_2mg/week** | **Lixisenatide_20 µg/day** |
| --- | --- | --- | --- |
| **Control** | -1.20 (-2.83 to 0.43) | -0.20 (-1.00 to 0.60) | -0.00 (-0.40 to 0.40) |
| 1.20 (-0.43 to 2.83) | **Exenatide_20 µg/day** | 1.00 (-0.82 to 2.82) | 1.20 (-0.48 to 2.88) |
| 0.20 (-0.60 to 1.00) | -1.00 (-2.82 to 0.82) | **Exenatide_2mg/week** | 0.20 (-0.70 to 1.10) |
| 0.00 (-0.40 to 0.40) | -1.20 (-2.88 to 0.48) | -0.20 (-1.10 to 0.70) | **Lixisenatide_20 µg/day** |

**Supplementary Table S7. League Table for PDQ-39**

| **Control** | **Exenatide_20 µg/day** | **Exenatide_2mg/week** | **Lixisenatide_20 µg/day** | **NLY01_2.5mg/week** | **NLY01_5mg/week** |
| --- | --- | --- | --- | --- | --- |
| **Control** | 3.50 ( -2.75 to 9.75) | -0.18 ( -2.42 to 2.06) | -0.30 ( -2.91 to 2.31) | -0.80 ( -2.30 to 0.70) | -0.60 ( -0.81 to -0.39) |
| -3.50 ( -9.75 to 2.75) | **Exenatide_20 µg/day** | -3.68 (-10.32 to 2.96) | -3.80 (-10.58 to 2.98) | -4.30 (-10.73 to 2.13) | -4.10 (-10.36 to 2.16) |
| 0.18 ( -2.06 to 2.42) | 3.68 ( -2.96 to 10.32) | **Exenatide_2mg/week** | -0.12 ( -3.56 to 3.32) | -0.62 ( -3.32 to 2.07) | -0.42 ( -2.67 to 1.83) |
| 0.30 ( -2.31 to 2.91) | 3.80 ( -2.98 to 10.58) | 0.12 ( -3.32 to 3.56) | **Lixisenatide_20 µg/day** | -0.50 ( -3.51 to 2.51) | -0.30 ( -2.92 to 2.32) |
| 0.80 ( -0.70 to 2.30) | 4.30 ( -2.13 to 10.73) | 0.62 ( -2.07 to 3.32) | 0.50 ( -2.51 to 3.51) | **NLY01_2.5mg/week** | 0.20 ( -1.30 to 1.70) |
| 0.60 ( 0.39 to 0.81) | 4.10 ( -2.16 to 10.36) | 0.42 ( -1.83 to 2.67) | 0.30 ( -2.32 to 2.92) | -0.20 ( -1.70 to 1.30) | **NLY01_5mg/week** |

**Supplementary Table S8. League Table for NMSS**

| **Control** | **Exenatide_2mg/week** | **NLY01_2.5mg/week** | **NLY01_5mg/week** |
| --- | --- | --- | --- |
| **Control** | 0.56 (-4.41 to 5.53) | 2.20 ( 1.76 to 2.64) | -0.60 (-1.05 to -0.15) |
| -0.56 (-5.53 to 4.41) | **Exenatide_2mg/week** | 1.64 (-3.35 to 6.63) | -1.16 (-6.16 to 3.83) |
| -2.20 (-2.64 to -1.76) | -1.64 (-6.63 to 3.35) | **NLY01_2.5mg/week** | -2.80 (-3.24 to -2.36) |
| 0.60 ( 0.15 to 1.05) | 1.16 (-3.83 to 6.16) | 2.80 ( 2.36 to 3.24) | **NLY01_5mg/week** |

**Appendix 5. Treatment rankings according to P-scores**

**Figure S15:** Ranking of Interventions Based on P-scores (MDS UPDRS Part 3 ON)


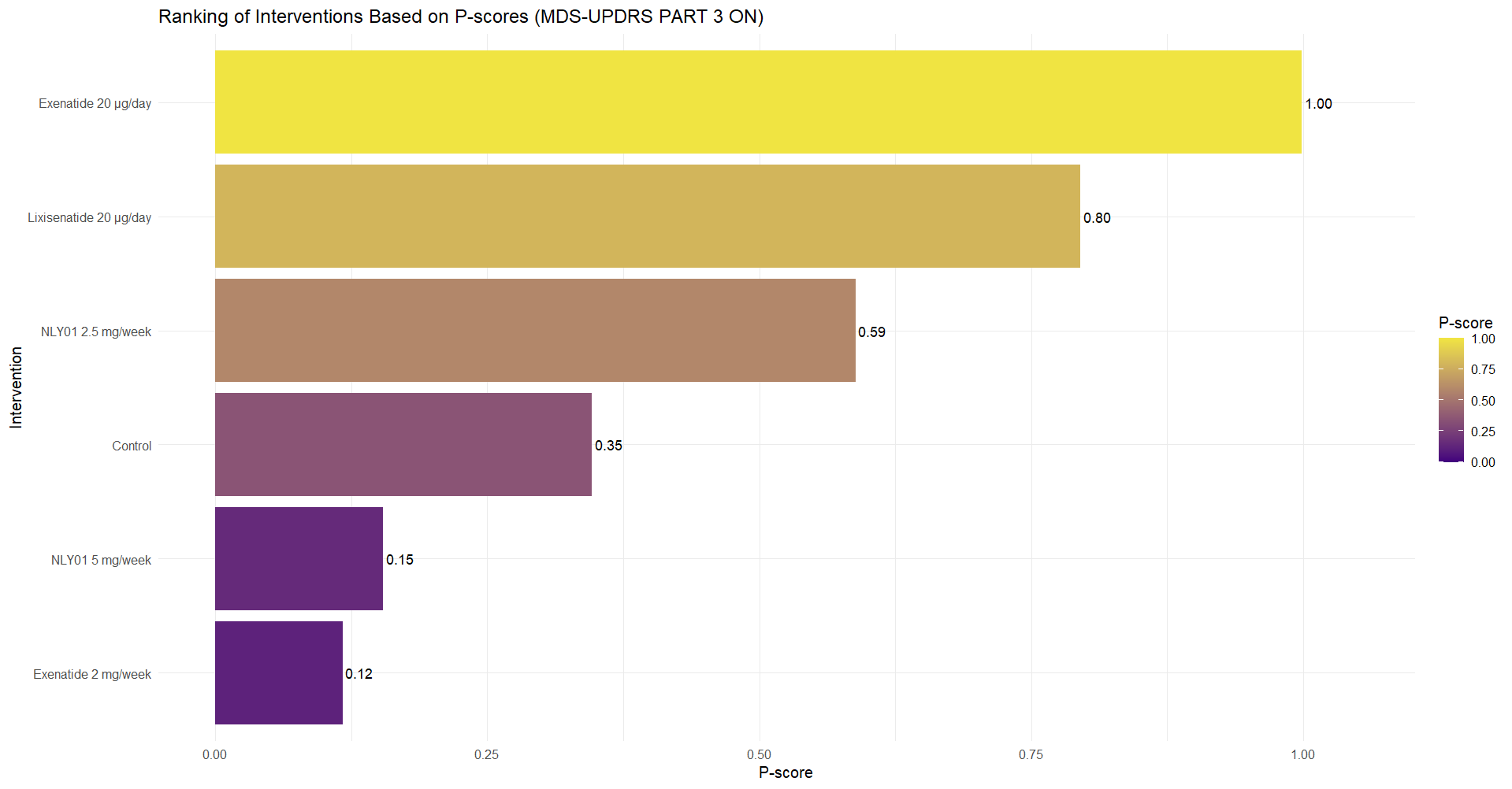


**Figure S16**: Ranking of Interventions Based on P-scores (MDS UPDRS Part 3 Off)


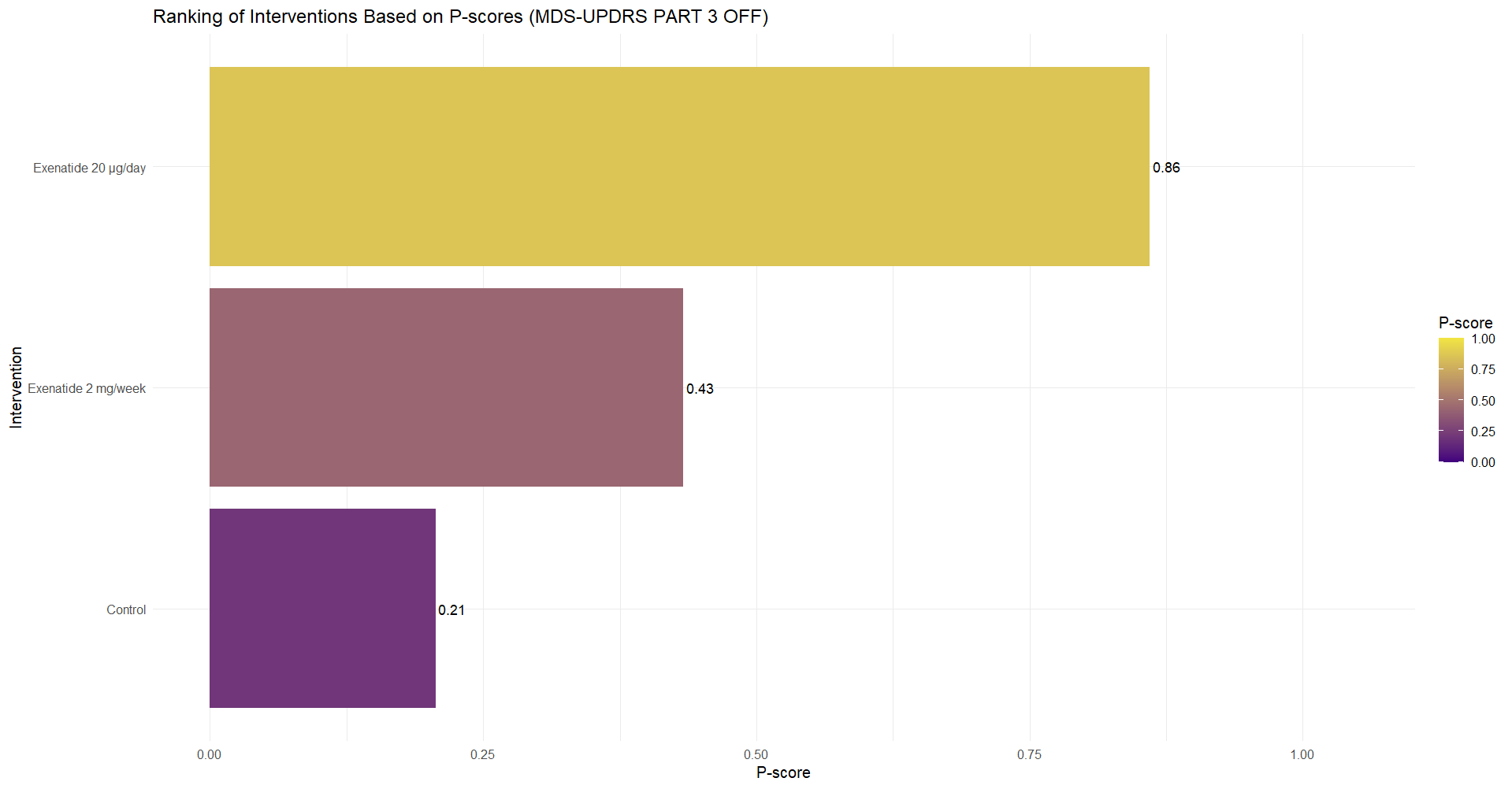


**Figure S17:** Ranking of Interventions Based on P-scores (MDS UPDRS Part 1 ON)


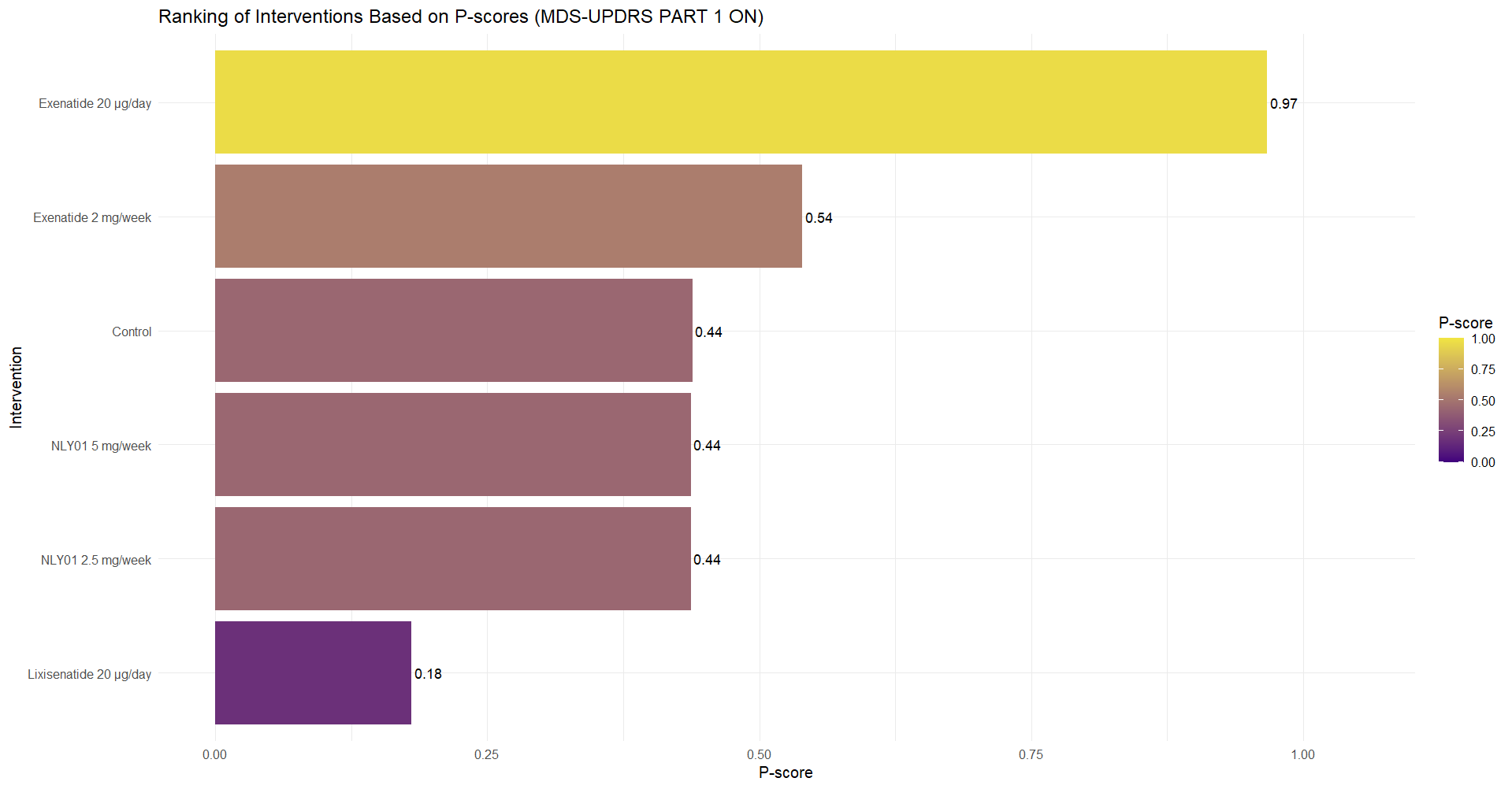


**Figure S18**: Ranking of Interventions Based on P-scores (MDS UPDRS Part 2 ON)


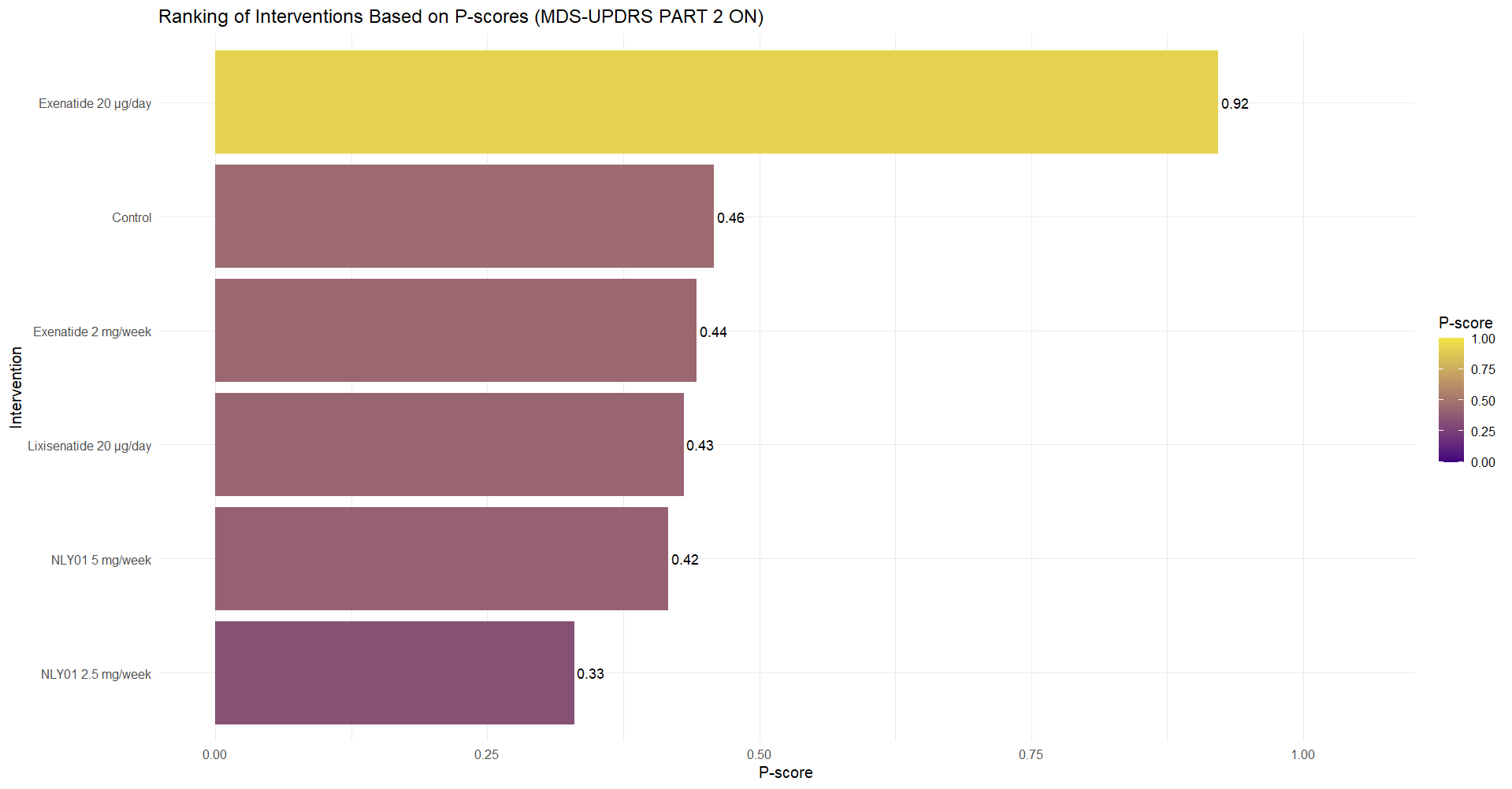


**Figure S19:** Ranking of Interventions Based on P-scores (MDS UPDRS Part 4 ON)


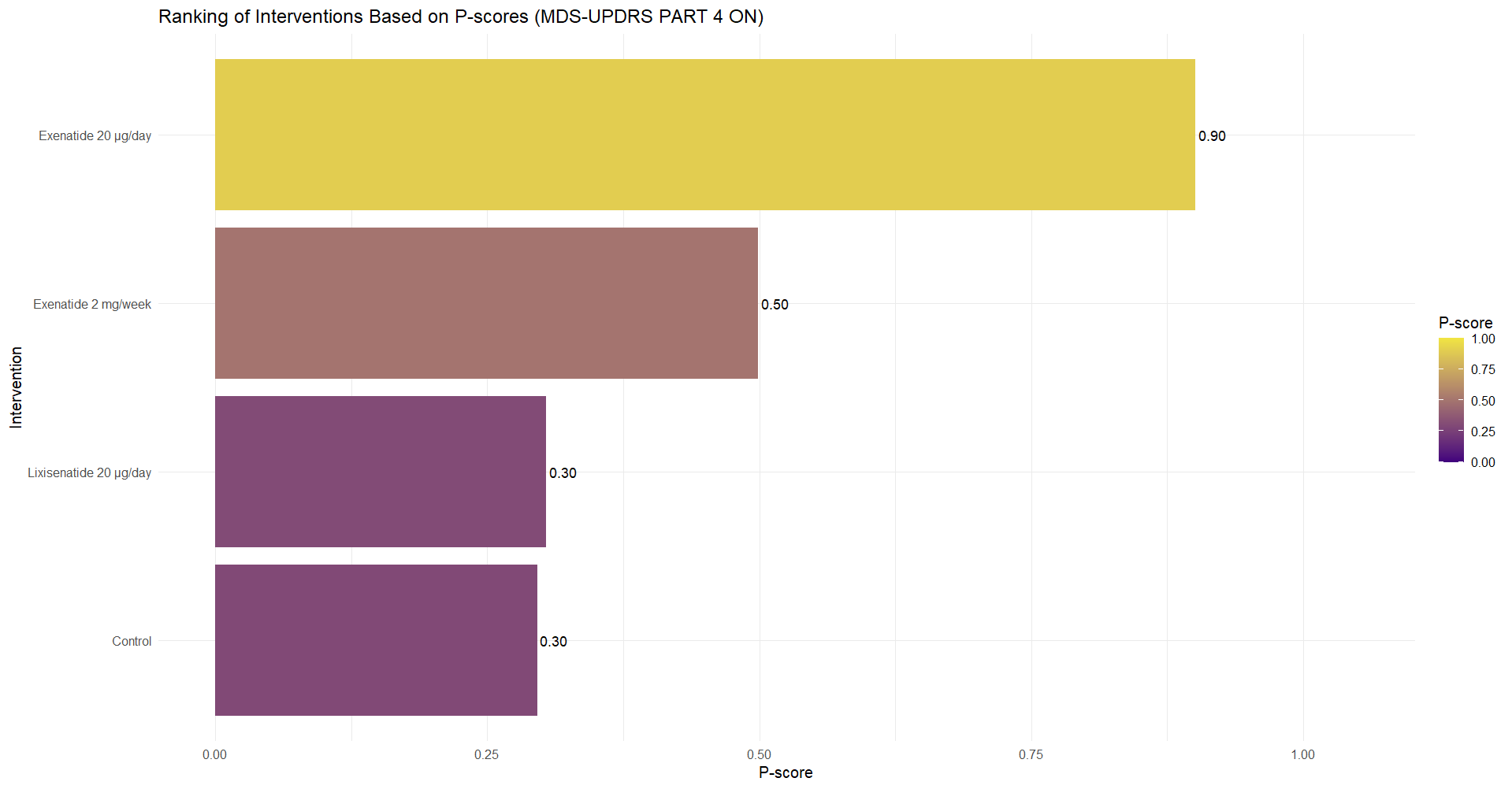


**Figure S20:** Ranking of Interventions Based on P-scores (PDQ-39)


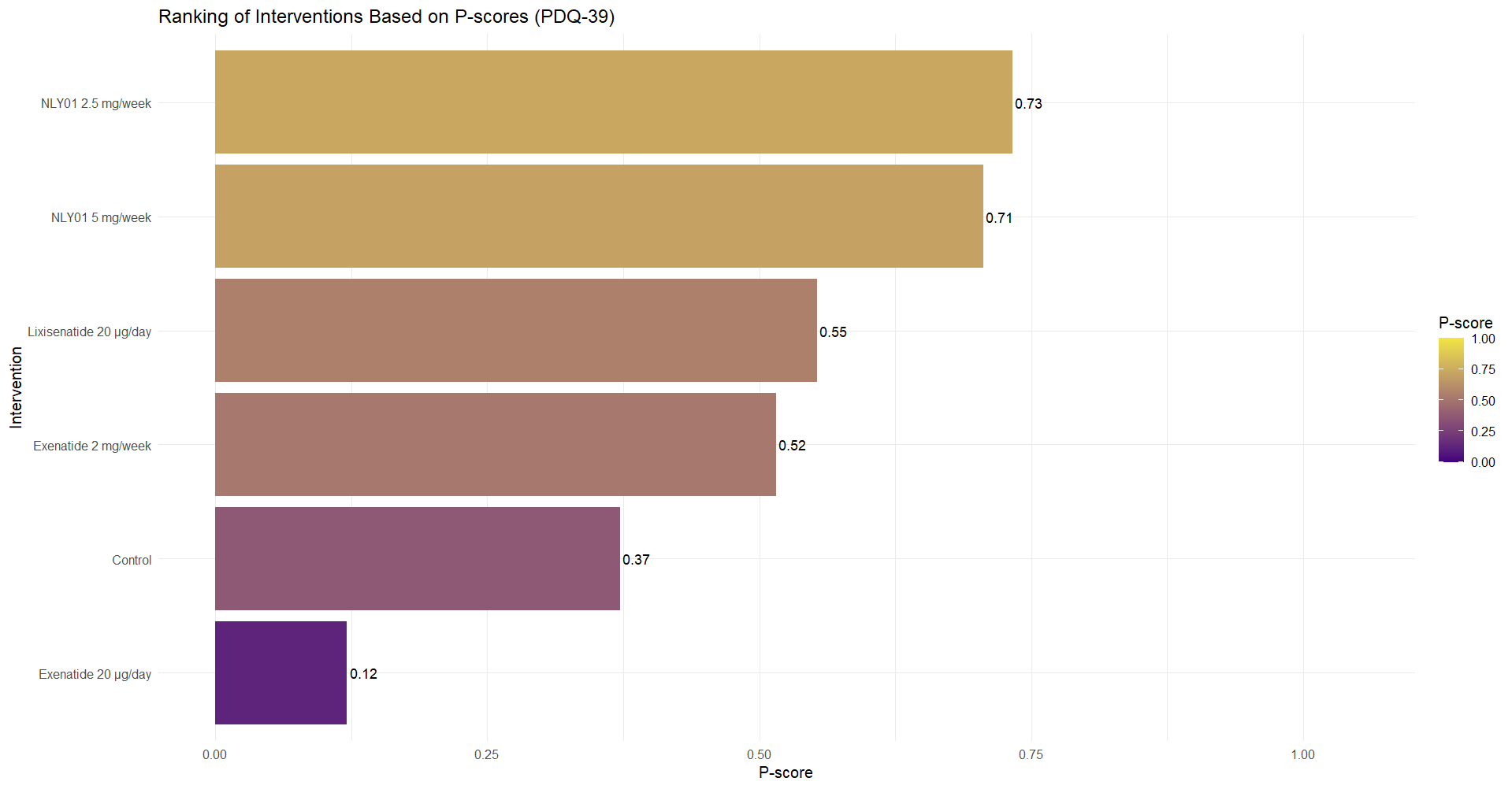


**Figure S21:** Ranking of Interventions Based on P-scores (NMSS)


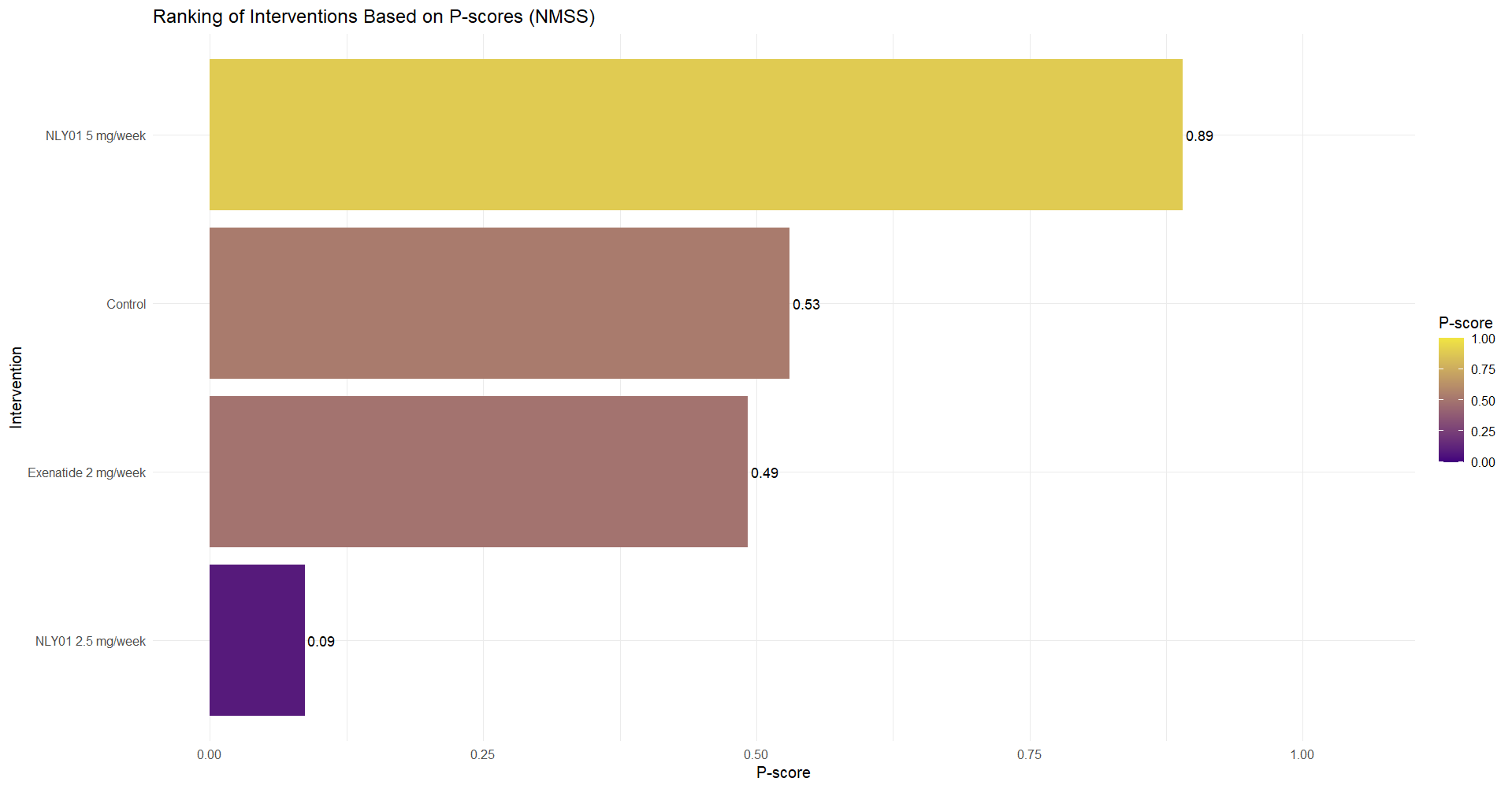


**Figure S22:** Risk Ratio of Fatigue


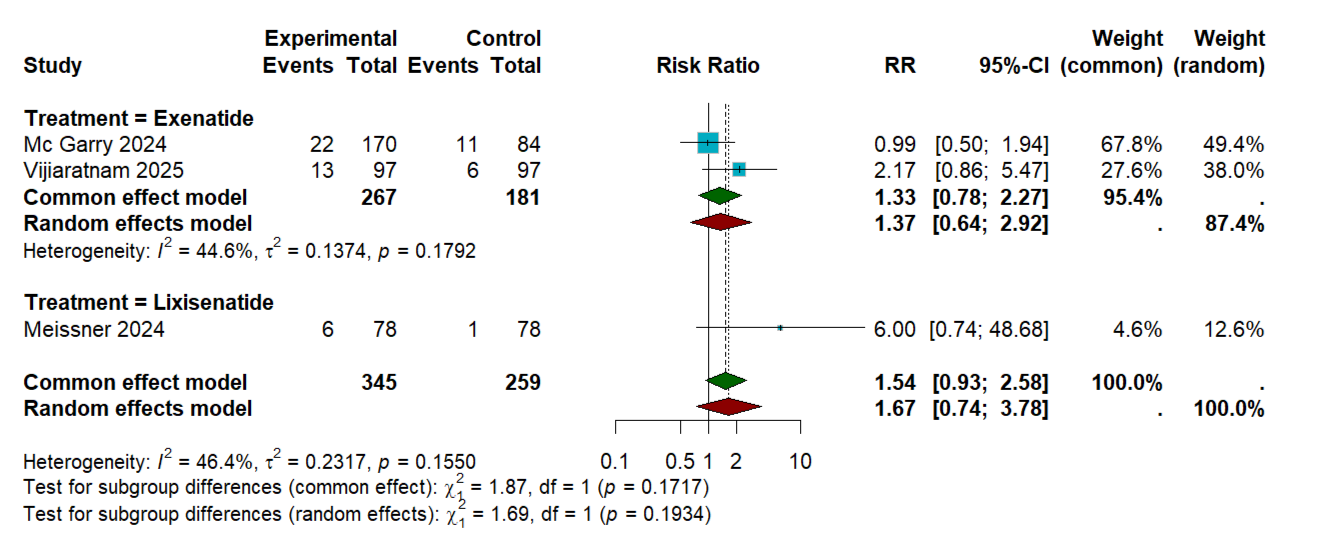


**Figure S23:** Risk Ratio of Anxiety


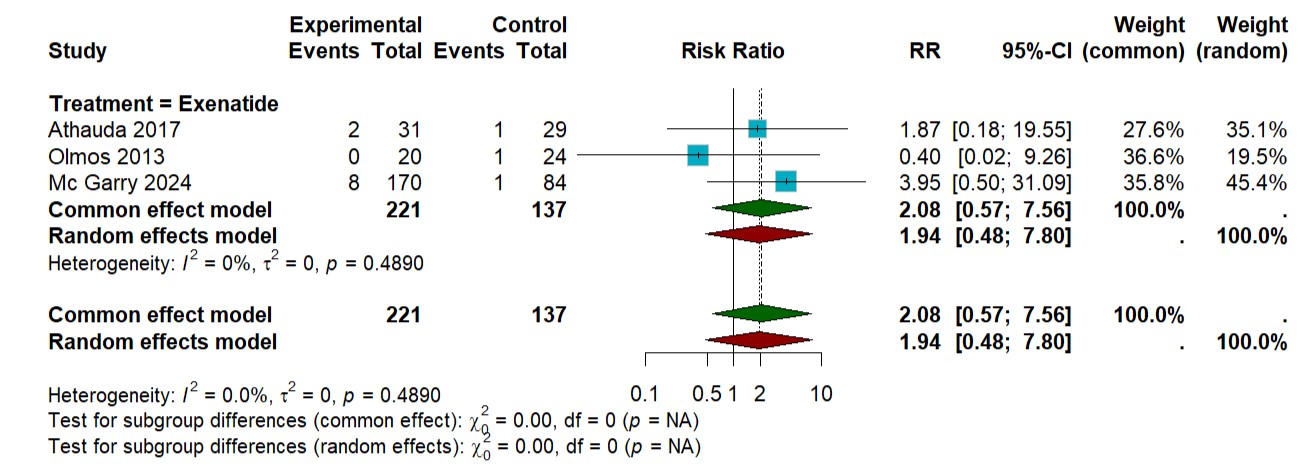


**Figure S24:** Risk Ratio of Constipation


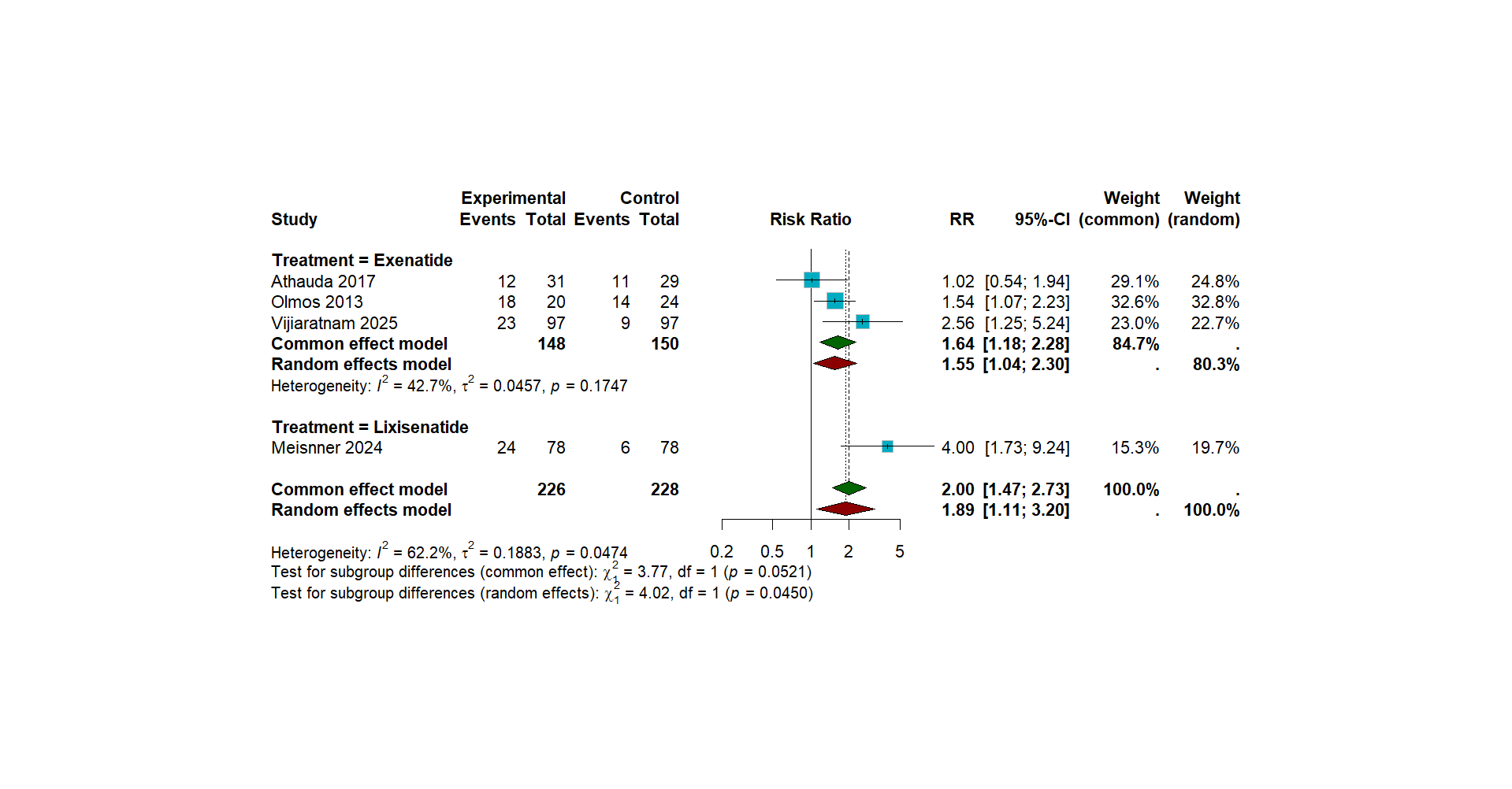


**Figure S25:** Risk Ratio of Diarrhea


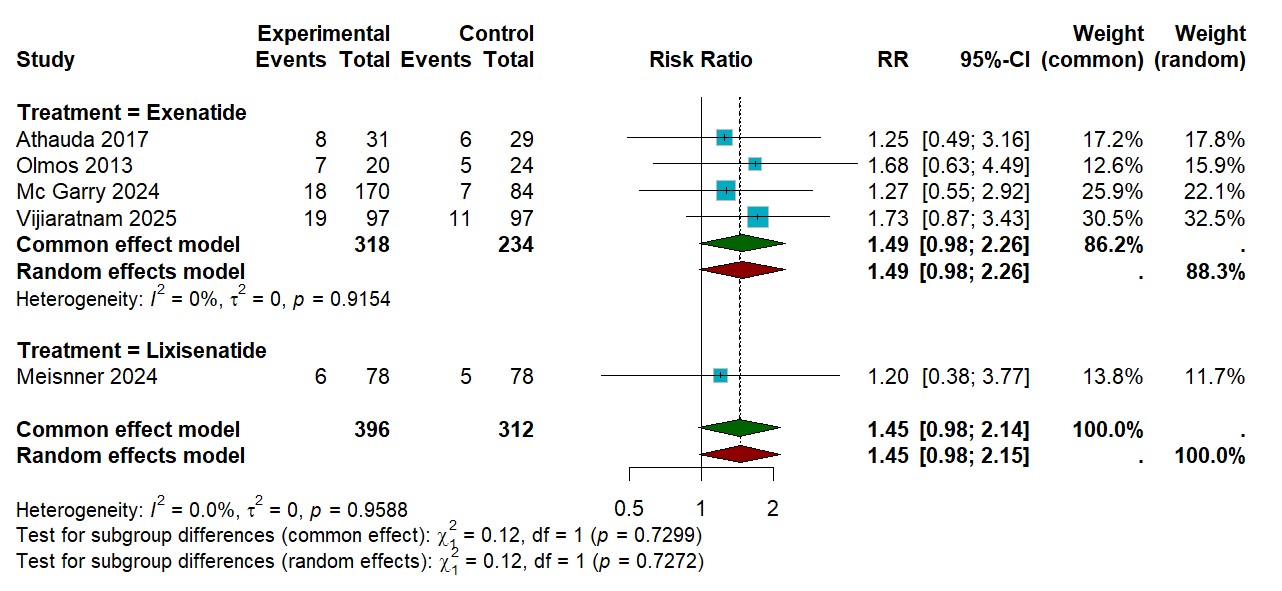


**Figure S26:** Risk Ratio of Headache


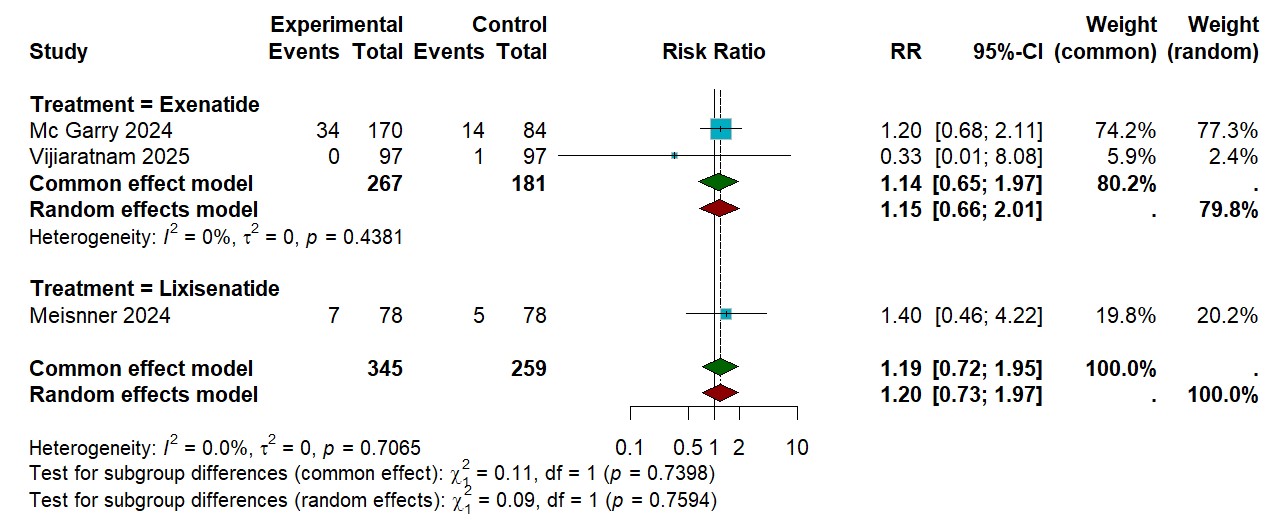


**Figure S27:** Risk Ratio of Nausea


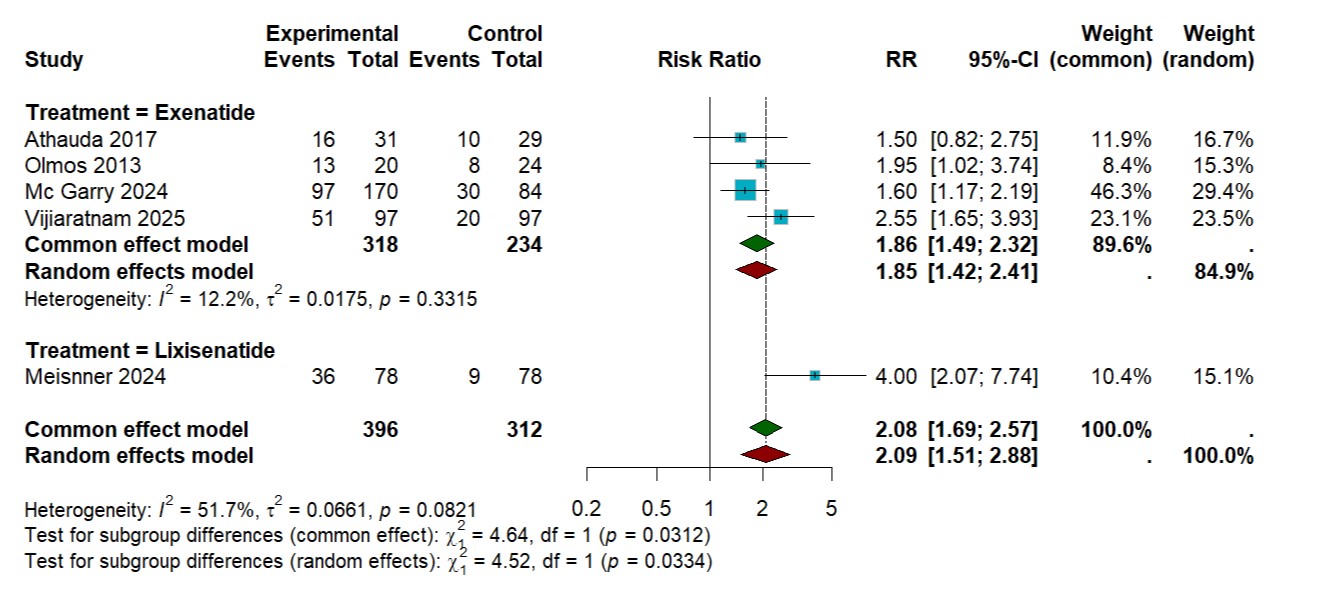


**Figure S28:** Risk Ratio of UTI


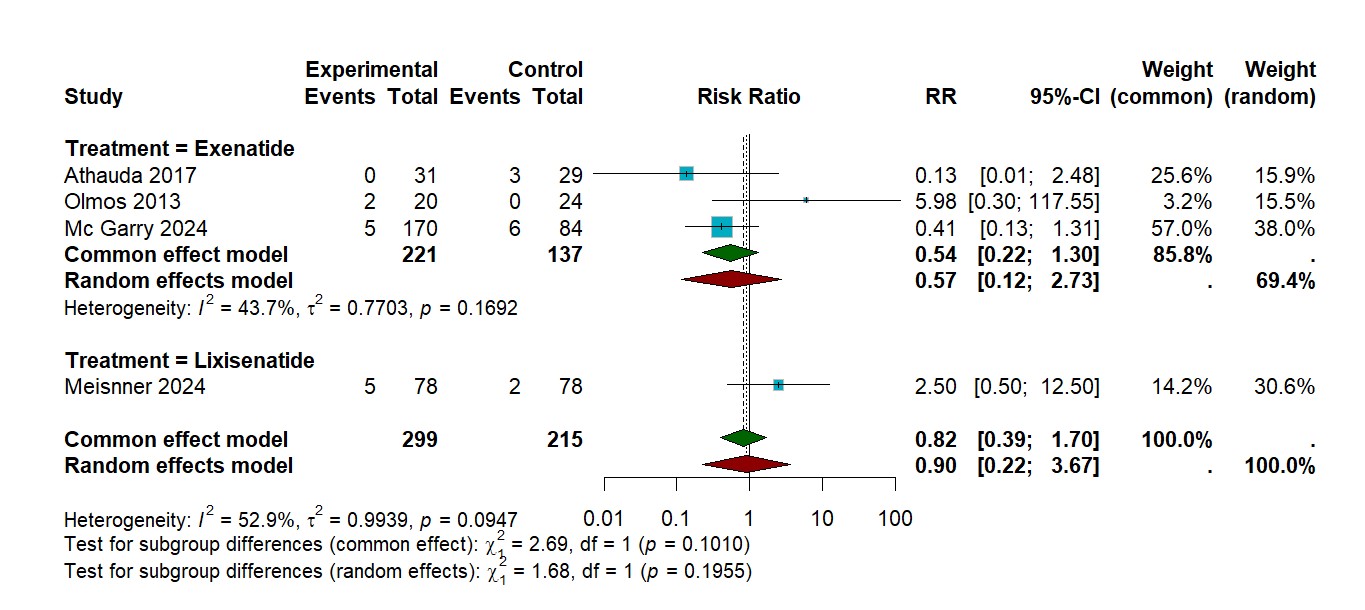


**Figure S29:** Risk Ratio of Vomiting


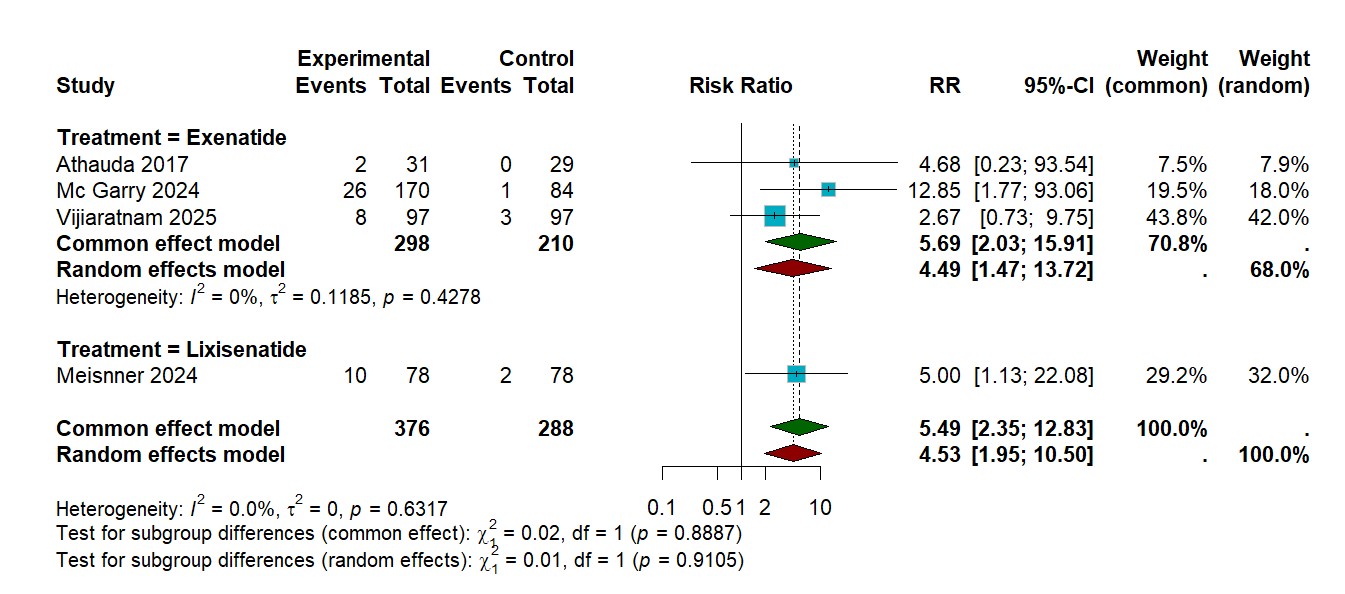


**Figure S30:** Risk Ratio of Weight Loss


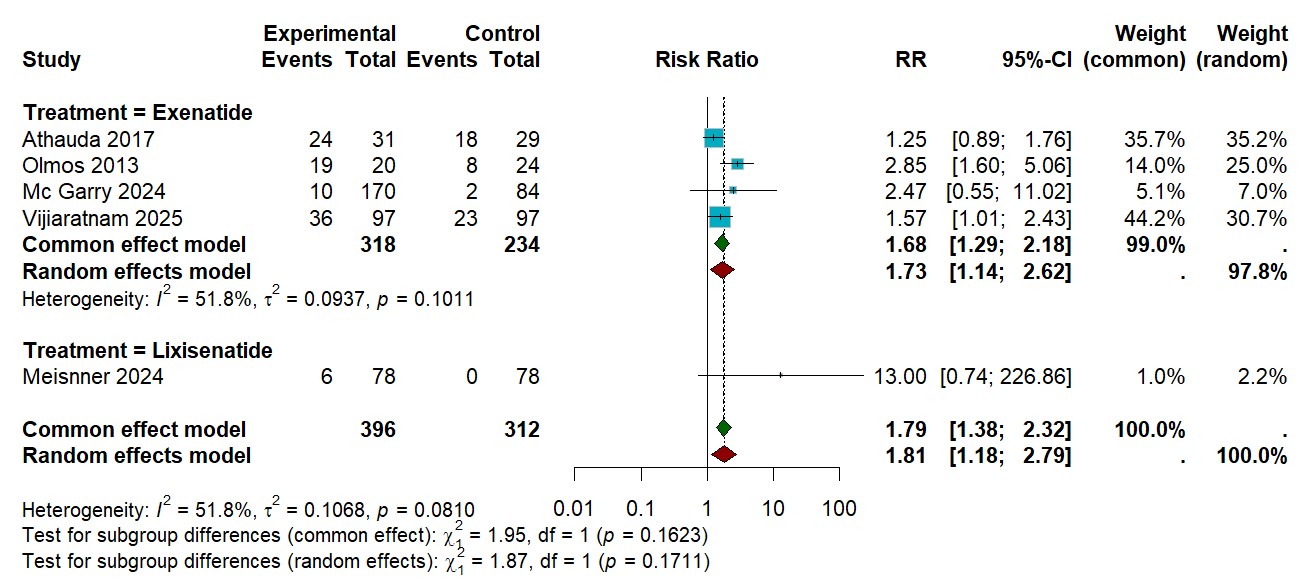


**Figure S31:** Risk Ratio of Administration site disorder


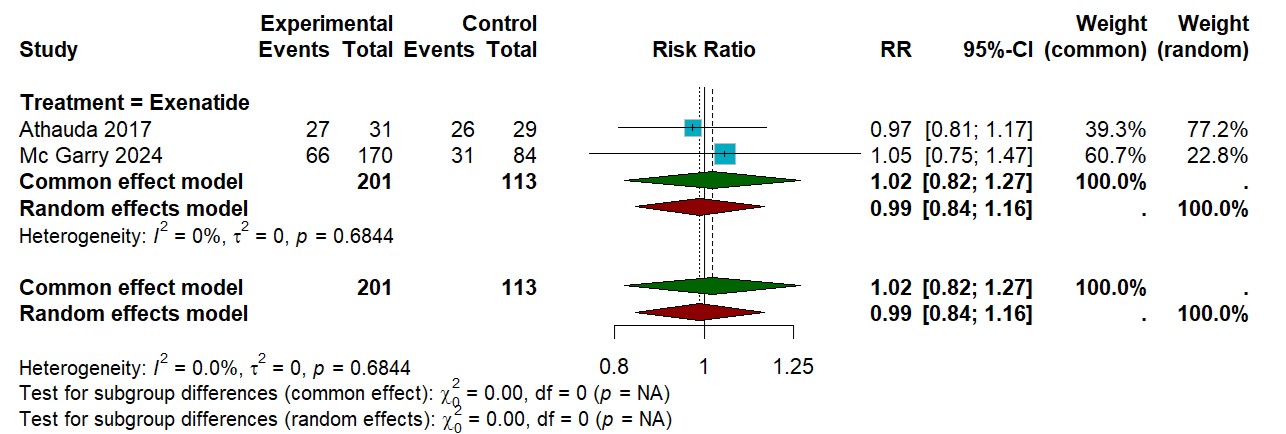

Supplement: Supplementary file 1 — Supplementary Material 1 [file 10072_2026_8929_MOESM1_ESM.docx]
